# Generation of Double-Labeled Reporter Cell Lines for Studying Co-Dynamics of Endogenous Proteins in Individual Human Cells

**DOI:** 10.1371/journal.pone.0013524

**Published:** 2010-10-21

**Authors:** Irina Issaeva, Ariel A. Cohen, Eran Eden, Cellina Cohen-Saidon, Tamar Danon, Lydia Cohen, Uri Alon

**Affiliations:** Department of Molecular Cell Biology, Weizmann Institute of Science, Rehovot, Israel; University of Colorado, Boulder, United States of America

## Abstract

Understanding the dynamic relationship between components of a system or pathway at the individual cell level is a current challenge. To address this, we developed an approach that allows simultaneous tracking of several endogenous proteins of choice within individual living human cells. The approach is based on fluorescent tagging of proteins at their native locus by directed gene targeting. A fluorescent tag-encoding DNA is introduced as a new exon into the intronic region of the gene of interest, resulting in expression of a full-length fluorescently tagged protein. We used this approach to establish human cell lines simultaneously expressing two components of a major antioxidant defense system, thioredoxin 1 (Trx) and thioredoxin reductase 1 (TrxR1), labeled with CFP and YFP, respectively. We find that the distributions of both proteins between nuclear and cytoplasmic compartments were highly variable between cells. However, the two proteins did not vary independently of each other: protein levels of Trx and TrxR1 in both the whole cell and the nucleus were substantially correlated. We further find that in response to a stress-inducing drug (CPT), both Trx and TrxR1 accumulated in the nuclei in a manner that was highly temporally correlated. This accumulation considerably reduced cell-to-cell variability in nuclear content of both proteins, suggesting a uniform response of the thioredoxin system to stress. These results indicate that Trx and TrxR1 act in concert in response to stress in regard to both time course and variability. Thus, our approach provides an efficient tool for studying dynamic relationship between components of systems of interest at a single-cell level.

## Introduction

Studying the dynamical relationship between different components of a system or pathway is crucial for understanding how proteins work together to generate cellular responses. A measurement system for such studies needs to follow dynamical changes in expression and localization of several proteins of interest over time in the same individual cells. Working at the level of individual cells is important due to cell-cell variability [1], [2], [3], [4], which is masked in assays based on averaging cell populations. Furthermore, averaging approaches can miss some dynamical features of protein behaviors, such as all-or-none effects [5] and oscillations [6], [7], [8], [9], as well as events that occur in only a subset of cells [10], [11].

Quantitative time-lapse fluorescence microscopy offers the advantage of tracking proteins in individual living cells over time [12], [13]. It requires usage of noninvasive fluorescent markers such as genetically encoded fluorescent proteins. Proteins labeled with a fluorescent tag tend to preserve the same half-lives [14], [15], [16], dynamics and localizations [10], [14], [15], [17], [18], [19] as their wild-type counterparts. Multicolor time-lapse imaging of two or more proteins, each tagged with a different fluorescent marker, provides a powerful tool to determine functional relationships between proteins within individual cells. Regulatory interactions can be revealed by analyzing dynamic correlations in gene expression fluctuations [20]. Spatial relationships between proteins within particular subcellular compartments can be detected by co-localization analysis, FRET and other approaches [21], [22], [23]. Multicolor live cell imaging is also especially useful for co-localization analysis of soluble proteins, since it overcomes potential concerns associated with cell fixation conditions [24]. Fluorescent tagging of proteins at the endogenous gene loci (rather than exogenous expression) is advantageous, because it preserves the native regulation of protein expression and avoids over-expression concerns. In the present context, we aimed to generate human reporter cell lines simultaneously expressing two endogenous proteins of interest, each labeled with a different fluorescent tag. Development of such multicolor reporter lines is challenging due to constraints of current approaches for genetic manipulations in human cells.

There are two major strategies for tagging endogenous proteins in mammalian cells. One is Central Dogma (CD) tagging. In this strategy, fluorescent tag-encoding DNA is introduced into genomic loci as a new exon. The tagging DNA is integrated into the genome in a random (non-directed) manner using a retrovirus [17], [25], [26], [27]. We previously reported the application of CD tagging to create a Library of Annotated Reporter Cells (LARC) in the H1299 human non-small lung carcinoma cell line [10], [25], [28]. Our LARC collection contains about 1200 cell clones, each expressing a different, annotated full-length protein labeled endogenously with a yellow fluorescent tag (YFP or Venus). It was used to study variability of protein levels between cells [1], [11], the prevalence of cell-cycle dependent protein dynamics [28], [29], and the effects of a cancer drug on the proteome [10]. Detailed information can be found in www.dynamicproteomics.net.

We also applied CD tagging to establish double-labeled reporter cells, in which one protein is tagged with YFP and the other with a red fluorophore, mCherry. This was achieved by two iterative rounds of CD tagging [1]. Since the combination of the two fluorescently labeled proteins in such reporter cells was based on the random retrovirus insertion, the approach is not feasible for targeting pre-selected genes.

The strategy that allows tagging proteins of interest is directed gene targeting. This is based on introducing into the cell exogenous DNA that contains regions of homology to the gene of interest flanking a selectable marker gene, which is integrated into the genome via homologous recombination. The most common application of directed targeting is inactivation of alleles of a gene of interest to generate knock-outs. The same strategy could also be exploited to introduce sequences into an endogenous gene, creating a knock-in allele. While routinely applied to diverse model organisms such as bacteria, yeast and rodents, application of directed gene targeting to human cells is much less efficient due to a lower rate of homologous recombination in human somatic cells [30], [31]. Recent advances in this area, such as utilization of recombinant Adeno-Associated Virus (rAAV) for delivery of targeting DNA [32], [33], [34] and use of promoterless configuration of selection markers [35], [36] made gene targeting in human cells more feasible. Up to date, tagging of endogenous proteins in human somatic cells by directed gene targeting has been performed primarily with short tag sequences, such as the FLAG epitope [37], [38]. Knock-in of large sequences, such as fluorescent protein sequences, has been used to report for endogenous promoter activity/gene expression [39] but not for protein tagging.

Here we present an approach for fluorescent labeling of endogenous proteins in human cells by directed gene targeting. In this approach, termed “directed CD tagging”, fluorescent protein-encoding DNA is introduced into desired genomic locus as an artificial exon. This exon is then spliced into the gene's mRNA to produce a full-length in-frame fusion protein. Iterative rounds of directed CD tagging using different fluorescent tags allow labeling several pre-selected proteins to study their dynamical relationship in vivo.

To test this strategy we chose the thioredoxin system [40], [41], [42], [43], [44], which plays a critical role in regulation of cellular redox homeostasis and cell defense against oxidative stress. This system is well studied, and both of its key proteins in single-tagged form are available in the LARC library. The main components of the system are oxidoreductase enzymes thioredoxin 1 (Trx) and thioredoxin reductase 1 (TrxR1). Trx reduces and thereby supports the activity of numerous proteins including ribonucleotide reductase, transcription factors and antioxidant peroxiredoxines. When reducing oxidized protein substrates, Trx itself becomes oxidized, and TrxR1 functions to recycle Trx back to the reduced (active) form. Both Trx and TrxR1 are ubiquitously expressed and reside predominately in cytosol. Upon exposure of cells to agents causing intracellular elevation of reactive oxygen species (ROS), Trx translocates from the cytosol into the nucleus [45], [46], [47], [48], [49], [50], [51], where it activates redox-sensitive transcription factors including NF-kappaB [52], [53], AP1 [54], p53 [55], Nrf2 [56], HIF [57] and others, which in turn activate expression of stress-responsive genes.

We applied directed CD tagging to label the Trx protein with a cyan fluorescent tag (CFP) in the LARC clone expressing the TrxR1-YFP fusion protein. The resulting double-labeled reporter cells allowed comparison of dynamical behaviors of Trx-CFP and TrxR1-YFP in individual living cells and were used to study the dynamics and cell-cell variability of the thioredoxin system. We found sizable variability across the cells in timing, expression and localization of the system's components. However, this variability was correlated between the two proteins, so that they seemed to vary together to a large extent. Correlation in spatio-temporal dynamics of the two proteins was most prominent in response to a drug-induced stress.

## Results

### Fluorescent labeling of endogenous proteins by directed CD tagging: a method overview

We combined ideas from CD tagging and directed gene targeting to develop a method for directed fluorescent labeling of endogenous proteins in human cells. We applied this to cells from the H1299 LARC library to generate double-labeled reporter cell lines. We used the LARC library, because it contains over a thousand clones expressing different endogenous YFP-labeled proteins. To create double-labeled cells, a pre-chosen LARC clone expressing a desired YFP-tagged protein was used as a parental line, onto which another protein of interest (e.g. from the same biological pathway) was tagged with a Cyan Fluorescent Protein (CFP) by directed gene targeting. For CFP-tagging we used the monomeric form of an improved variant of CFP, Cerulean [58]. For simplicity, monomeric Cerulean will be further referred to as CFP.

**Figure 1:**
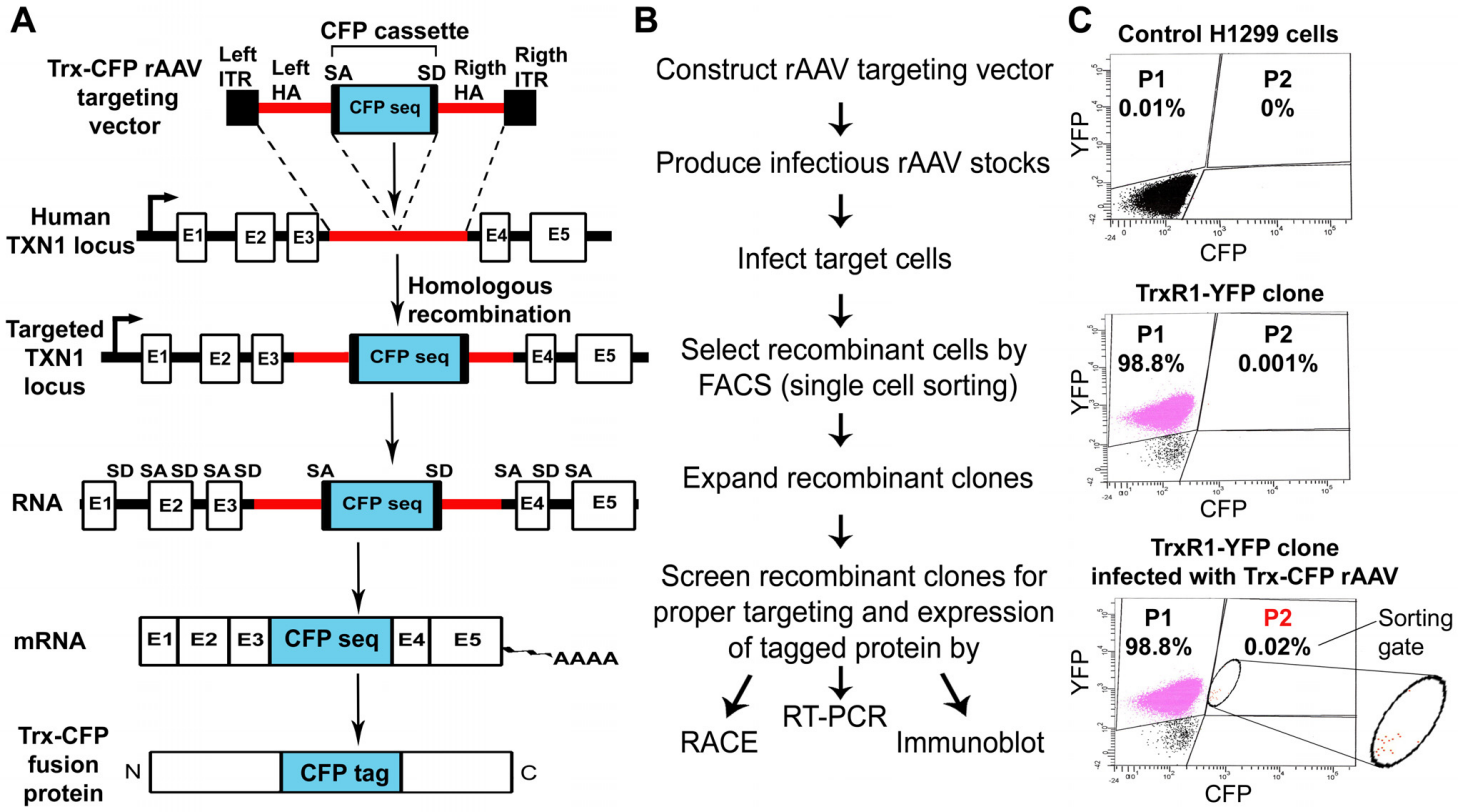
Fluorescent labeling of endogenous proteins by directed CD tagging. **A**) Schematic diagram of the tagging approach. Insertion of the CFP cassette into intron 3 of TXN1 is mediated by rAAV. Correct site-specific integration results in retaining CFP sequence as a new exon after the splicing of mRNA. The tagged mRNA translates to the internally labeled Trx protein. ITR, inverted terminal repeat; HA, homologous arm; SA, splicing acceptor, SD, splicing donor; CFP seq, CFP sequence; E, exon. **B**) Flow chart of the tagging procedure. **C**) Selection of recombinant (CFP-positive) cells by FACS. FACS histograms are shown for the control H1299 cells, the parental TrxR1-YFP clone and the TrxR1-YFP cells infected with Trx-CFP rAAV. The P1 region of the plot indicates the YFP-positive cells, the P2 region indicates the sorting gate used for selection of the CFP-positive portion of the infected TrxR1-YFP cells.

The tagging procedure is outlined in Figure 1B. The CFP-encoding DNA sequence is introduced into gene locus of interest by means of homologous recombination mediated by recombinant Adeno-Associated Virus (rAAV). As shown in Figure 1A, the CFP-tagging rAAV targeting construct contains two regions of homology to an intronic sequence of the target gene, flanking the CFP cassette. Similarly to CD-tagging, our targeting approach uses an artificial exon strategy: the CFP cassette in the targeting vector consists of the CFP-coding sequence (with no start and stop codons) flanked by splice acceptor and donor sequences. When integrated into the intronic region of the target gene, the artificial exon is spliced into the gene's mRNA, and a full-length fusion protein is translated (Figure 1A). Expression of the fluorescent tag from the native chromosomal location depends on production of an in-frame fusion protein. Thus, the fluorescent protein serves as both a reporter tag and a promoterless selection marker. The CFP-positive knock-in cells are selected by flow cytometric single-cell sorting. It should be noted that tagged cells generated in this way are heterozygous with respect to the CFP knock-in.

### CFP tagging of Trx in the TrxR1-YFP LARC clone

The directed CD tagging approach was applied to tag the Trx protein with CFP, in the LARC clone expressing the TrxR1-YFP fusion protein. The CFP-tagging rAAV vector was designed to target intron 3 of the thioredoxin 1 gene, TXN1 (Figure 1A).

The thioredoxin targeting vector was packaged into rAAV. The TrxR1-YFP cell line was then infected with the targeting virus, and CFP-expressing knock-in cells were selected by FACS. As seen in Figure 1C, the fraction of CFP-positive cells was about 0.02%. These cells were automatically collected by single cell sorting into individual wells of 384-well plates. Since the proportion of CFP-positive cells in the total population of infected cell is very low, the percentage of false-positive events introduced by FACS during the sorting stage is expected to be relatively high. Therefore, following FACS sorting the cells were further screened for CFP expression by fluorescence microscopy to detect true-positive events. Several independent sorting experiments were performed. Typically, out of 384 cells plated, about 100–120 (26–30%) survived the sorting stage and formed colonies. Among these colonies, 3–12 clones (3–10%) represented true-positive events and expressed CFP, as detected by fluorescence microscopy. To summarize, FACS sorting of ∼7–8×10^6^ infected cells (an equivalent of 50% confluent 15 cm plate) typically resulted in establishing of 3–12 CFP-positive (recombinant) clones. Thus, the overall efficiency of the tagging procedure in H1299 cells is about 1×10^−6^. The established recombinant clones were further expanded and screened for proper targeting.

To ascertain whether the CFP sequence in the recombinant clones was present only within the thioredoxin gene transcript, the identity of CFP labeled RNA was determined by rapid amplification of cDNA 3′end (3′ RACE) and subsequent sequencing. The CFP (mCerulean) primers utilized in the 3′ RACE analysis were chosen to avoid cross-reactivity with YFP. These primers, which spanned CFP (mCerulean) nucleotides 432–451 and 606–626, were used in the 1^st^ and 2^nd^ (nested) PCR amplifications, respectively (for details see Materials and Methods section).

**Figure 2:**
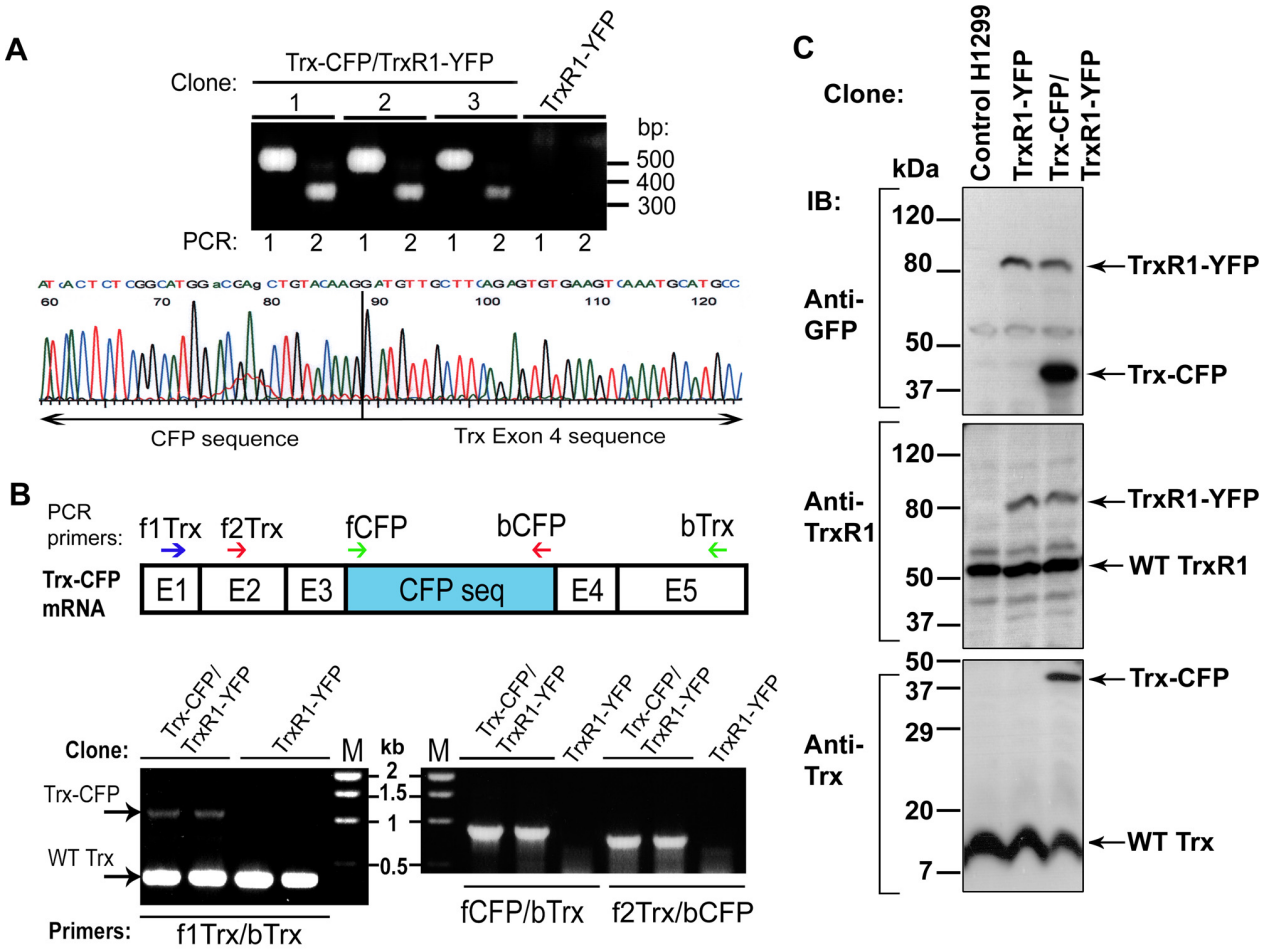
Test of proper targeting and expression of Trx-CFP. **A**) 3′RACE and sequencing. The upper panel shows 3′RACE results for three representative CFP-tagged clones (Trx-CFP/TrxR1-YFP) and for the control parental TrxR1-YFP cells. First lane (1) indicates first PCR and second lane (2) indicates the nested PCR for each clone. The PCR products obtained for the TrxR1-YFP/Trx-CFP clones were further subjected to sequencing. The lower panel shows a fragment of representative sequencing chromatogram. The correct fusion of the 3′ end of CFP sequence to the beginning of the Trx exon 4 is indicated. **B**) RT-PCR analysis. The CFP-tagged Trx mRNA is schematically depicted on the upper panel. Positions of the primers used for RT-PCR are designated by arrows. The lower panel shows representative RT-PCR results for two Trx-CFP/TrxR1-YFP clones and for the parental TrxR1-YFP clone. The primer pairs used for each reaction are indicated in the bottom. Positions of the PCR products corresponding to the wild-type (non-tagged) and to the CFP-tagged Trx mRNA are denoted by arrows. **C**) Immunoblot analysis. The control H1299 cells, the parental TrxR1-YFP clone and the Trx-CFP/TrxR1-YFP double-labeled cells were immunoblotted (IB) with anti-GFP,-TrxR1 and -Trx antibodies. Bands corresponding to the wild-type and tagged proteins are marked by arrows.

More than 20 recombinant clones were screened by 3′ RACE. Representative results are shown in the upper panel of Figure 2A. While no PCR products were observed with the parental TrxR1-YFP clone, for all recombinant clones, the 1^st^ and the 2^nd^ PCR reactions uniformly yielded single-band products. The band of the nested reaction was lower than that of the 1^st^ PCR by a nesting distance of 174 bp. These results demonstrate that all recombinant clones tested expressed only one CFP-tagged gene transcript. Subsequent sequencing of 3′RACE products further confirmed that this transcript corresponds to Trx mRNA. Sequencing data also confirmed a precise in-frame fusion of the CFP 3′ end to the exon 4 of Trx mRNA (a fragment of typical chromatogram displaying the 3′ fusion point is shown in Figure 2A, lower panel). The correct fusion at the mRNA level indicates that the artificial exon CFP cassette has been properly integrated into intron 3 of the TXN1 gene.

Trx targeting was further validated at the mRNA level by RT-PCR. Two screening approaches were used. The first employed one PCR primer complementary to CFP and the second complementary to the Trx exonic sequence located either upstream or downstream of CFP (the primer positions are schematically depicted in Figure 2B, upper panel). When using these primers, the PCR product is amplified only from the CFP-tagged Trx mRNA. Indeed, as shown in Figure 2B (right lower panel), a single dominant PCR band of correct size was observed in the recombinant clones, but not in the parental TrxR1-YFP cells. The second RT-PCR screening approach utilized the primers' pair complementary to the first and to the last exon of Trx. This approach resulted in amplification of two PCR products in recombinant clones: a 414 bp-long band corresponding to the wild-type (non-tagged) Trx mRNA and a 1128 bp-long band corresponding to the Trx mRNA tagged with CFP (the CFP sequence is 714 bp in size). In the parental TrxR1-YFP clone only a 414 bp product was amplified (Figure 2A, left lower panel).

To summarize, 3′RACE and RT-PCR analyses demonstrated that CFP-tagging was unique, specific and correct in all clones tested. Thus, our method can readily yield productive cell lines from rare but precise recombination events.

### Validation of expression of the Trx-CFP and TrxR1-YFP fusion proteins in Trx-CFP/TrxR1-YFP reporter cells

The expression of full-length Trx-CFP and TrxR1-YFP fusion proteins in Trx-CFP/TrxR1-YFP double-labeled reporter cells was further tested by immunoblotting. Molecular weights of the non-tagged TrxR1 and Trx proteins are ∼55 kDa and ∼13 kDa, respectively. Addition of the YFP/CFP tag increases the proteins' weight by ∼27–30 kDa. As shown in Figure 2C (upper panel), the full-length TrxR1-YFP and Trx-CFP fusion proteins (MW ∼84 kDa and ∼42 kDa, respectively) were recognized by anti-GFP antibody. Anti-TrxR1 and anti-Trx antibodies also recognized the corresponding fusion protein, together with its wild-type counterpart expressed from the untagged alleles (Figure 2C, the middle and the lower panels). Immunoblot analysis with protein-specific antibodies indicated that the protein bands corresponding to either TrxR1-YFP or Trx-CFP were about 2-fold less intense than those corresponding to the non-tagged proteins. This suggests that the tagged and non-tagged TrxR1 and Trx are expressed at a ratio of about 1∶2. These results are consistent with previous kariotyping data which showed that parental H1299 cells have three copies of both chromosome 12, containing the thioredoxin reductase 1 gene, and chromosome 9, containing the thioredoxin1 gene (data not shown), of which only one copy is expected to be tagged. These results suggest that both TrxR1 and Trx proteins are expressed from the tagged allele at about the same level as from the untagged alleles.

### Quantification of Trx-CFP and TrxR1-YFP in individual living cells reveals high variability in nuclear levels of both proteins

**Figure 3:**
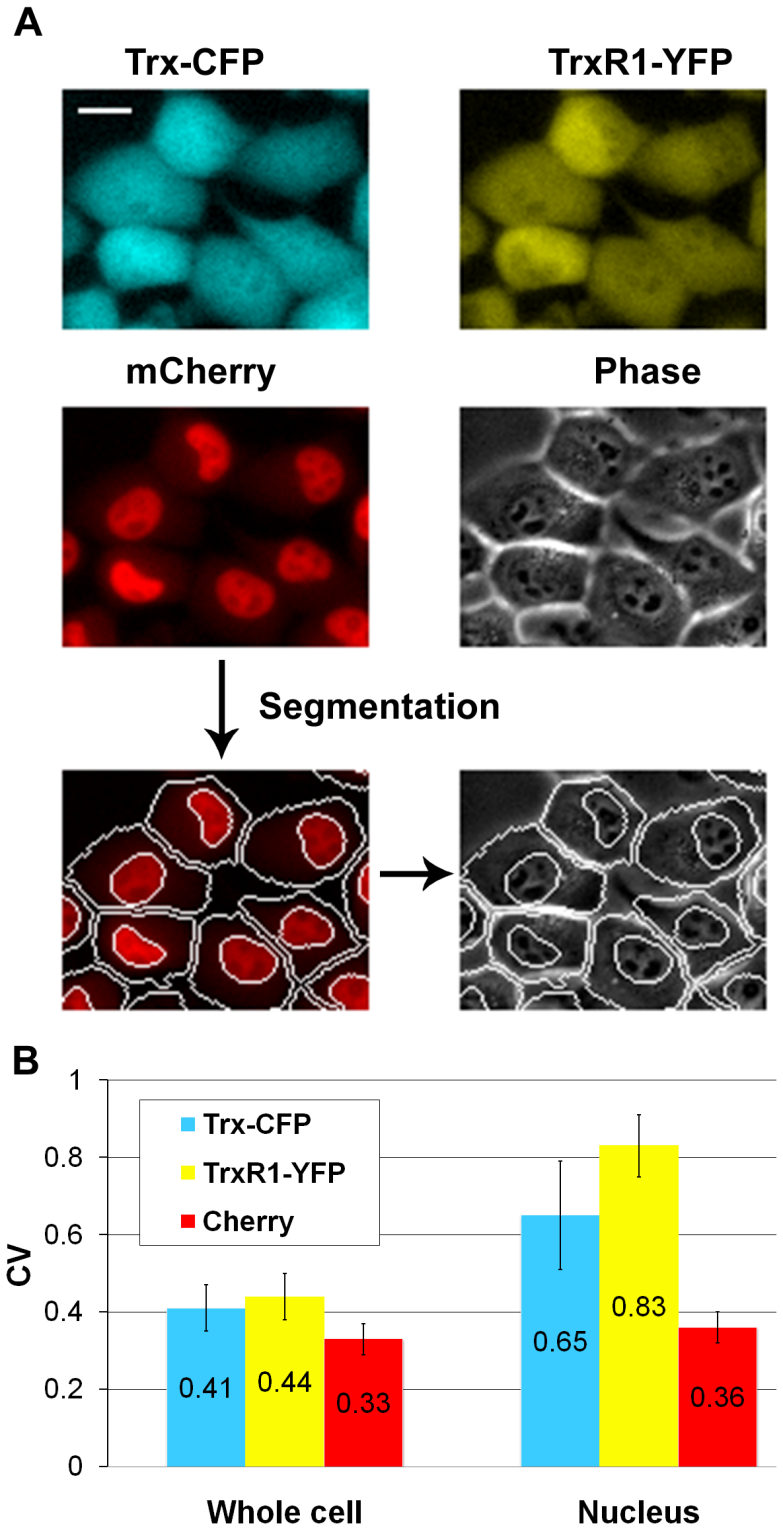
Individual cell measurements show high variability in nuclear levels of Trx-CFP and TrxR1-YFP. **A**) Fluorescent and phase-contrast images of Trx-CFP/TrxR1-YFP reporter cells. mCherry fluorescence is used for automated detection of cellular and nuclear boundaries. Scale bar denotes 20 microns. **B**) The cell-to-cell variability in Trx-CFP and TrxR1-YFP nuclear levels is significantly higher than the variability observed for the whole cell protein content. The coefficient of variance (CV) represents the ratio between standard deviation and mean. Error bars denote standard error.

To determine protein levels and localizations of Trx-CFP and TrxR1-YFP in individual living cells we employed time-lapse fluorescence microscopy under controlled CO2, temperature and humidity, as described in Cohen *et al.* [10]. Fluorescent images were analyzed using automated image analysis software (see Materials and Methods). Cell segmentation was achieved by means of a third fluorophore, mCherry (red). This tag was introduced by CD tagging into parental H1299 cells prior to the LARC library construction, and is common to all LARC clones. mCherry is integrated into genomic sequences of two proteins: XRCC5, localized to the nucleus, and DAP1, localized mainly to the cytoplasm. Expression of these two tagged proteins creates a mCherry fluorescence pattern which is bright in the nucleus and dimmer in the cytosol. This pattern allows automated detection of cellular and nuclear boundaries (Figure 3A, lower panel) [10].

As shown in Figure 3A (upper panel), Trx-CFP and TrxR1-YFP are localized to both cell nuclei and cytoplasm. This is consistent with subcellular distribution of Trx and TrxR1 reported for non-small cell lung carcinomas [59].

Image analysis allowed accurate quantification of Trx and TrxR1 protein levels in the whole cell and in the nucleus, defined as the total CFP/YFP fluorescence within the cell and nuclear boundaries. On average, the nuclear fraction of Trx-CFP and TrxR1-YFP comprised about 43% and 39%, respectively, of the total protein levels. Variability was observed in both the levels and subcellular distributions of Trx-CFP and TrxR1-YFP across individual cells. The variability in total protein levels of Trx-CFP and TrxR1-YFP was comparable to that of the Cherry-tagged proteins (Figure 3B). The average ratio between fluorescence of the 90^th^ percentiles of the cells to the 10^th^ percentiles of the cells (90∶10 ratio) was 2.3±0.3 for Trx-CFP, 2.4±0.3 for TrxR1-YFP and 2.1±0.24 for Cherry. Similarly, the average coefficient of variation (CV = standard deviation/mean) was 0.41±0.08 for Trx-CFP, 0.44±0.06 for TrxR1-YFP and 0.35±0.04 for Cherry (Figure 3B). This level of variability is comparable to that found in other proteins in human cells [1], [11]. Variability in nuclear levels of both Trx-CFP and TrxR1-YFP was considerably higher than that of Cherry (Figure 3B). Thus, the average 90∶10 ratio of nuclear fluorescence was 3.1±0.6 and 3.8±0.4, for Trx-CFP and TrxR1-YFP respectively, and 2.2±0.23 for Cherry. CV of nuclear fluorescence for Trx-CFP and TrxR1-YFP was 0.65±0.14 and 0.75±0.08, respectively, and these values were about 2-fold higher than the CV of Cherry (Figure 3B). This heterogeneity in nuclear levels of Trx-CFP and TrxR1-YFP is clearly observable by eye and stems mainly from cells that are highly enriched in nuclear Trx-CFP and TrxR1-YFP relative to the rest of the population (Figure S1). In line with this observation, presence of a cell fraction enriched in nuclear Trx has been recently shown for the HUVEC cell line using immunostaining of fixed cells [60].

Both total and nuclear levels of Trx-CFP and TrxR1-YFP within individual cells were substantially correlated. The correlation between Trx-CFP and TrxR1-YFP is discussed below.

### Trx and TrxR1 localize to the nucleus in response to CPT

It has been previously shown that exposure of cultured cells to agents causing elevation of intracellular reactive oxygen species (ROS) results in translocation of Trx from the cytoplasm to the nucleus [47], [49], [51], [53]. This translocation is suggested to be a hallmark of oxidative stress response [51].

Generation of Trx-CFP/TrxR1-YFP reporter cells allows studying the dynamical relationship between components of the thioredoxin system within the same cells during their response to oxidative stress. For such experiments we chose to use camptothecin (CPT). CPT evokes oxidative stress [61], [62], has been used in our previous studies of proteome' dynamics, and was shown to induce nuclear translocation of both TrxR1 and Trx, present in the LARC library as YFP fusion proteins [10].

Using automated time-lapse fluorescence microscopy we followed dynamics of Trx-CFP and TrxR1-YFP in individual cells upon addition of 10 µM CPT to the cell medium. Time-lapse movies were acquired for a period of 30–40 hours, at a temporal resolution of 20 min. Each movie allowed tracking of individual cells from 48 fields of view; each field of view contained 50–100 cells (for more details see Material and Methods section). Relative error of the average fluorescence measurements between day-to-day repeats was about 15%. The protein response to CPT was analyzed for the first 25 hours; after this time point accumulation of dead cells precluded precise analysis.

**Figure 4:**
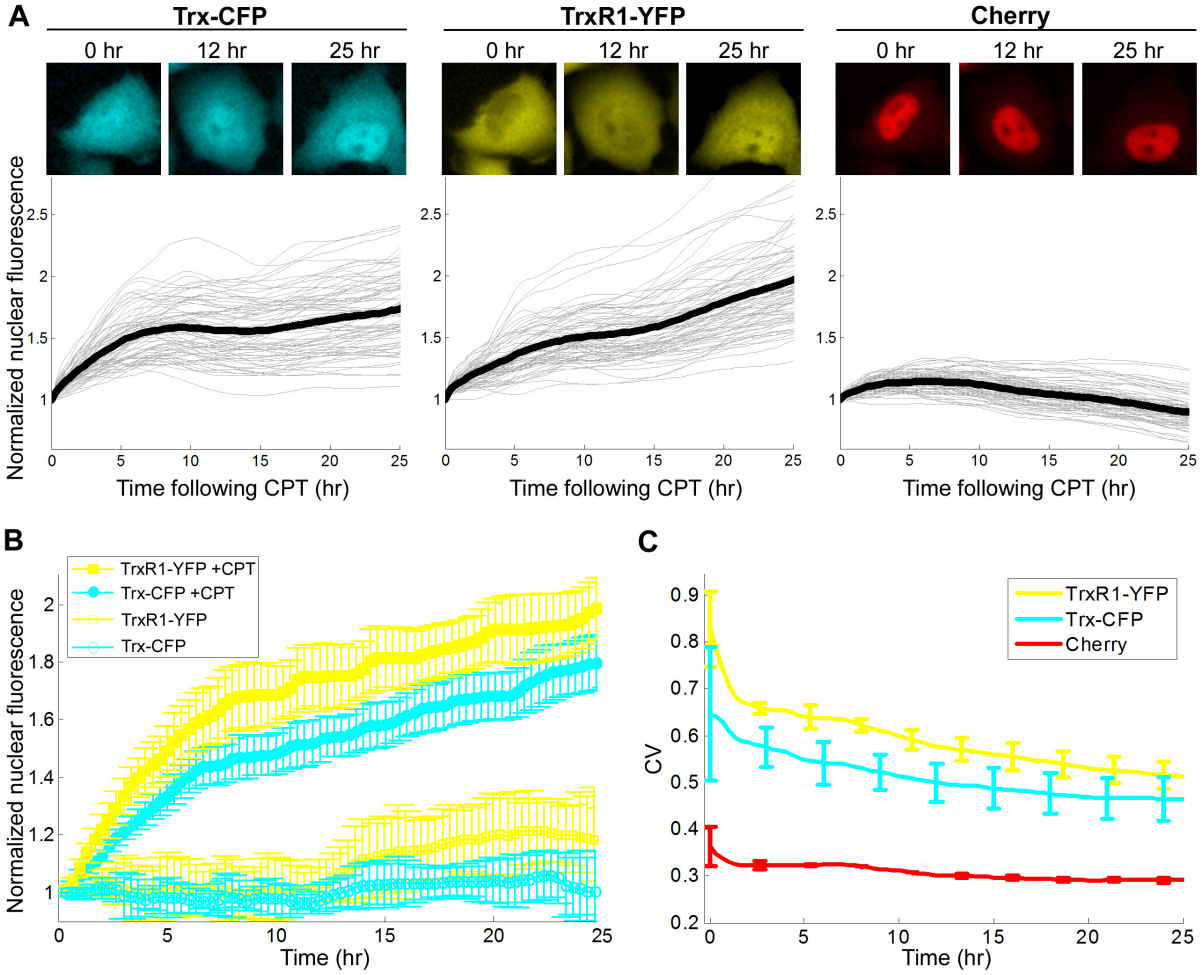
Nuclear accumulation of Trx-CFP and TrxR1-YFP in response to CPT. **A**) Nuclear fluorescence profile of Trx-CFP, TrxR1-YFP and Cherry following CPT treatment. The upper panel shows fluorescent images of a representative cell expressing Trx-CFP and TrxR1-YFP. Time after CPT addition is indicated above each image. The lower panel: thin lines denote nuclear fluorescence of individual cells normalized to their initial nuclear fluorescence prior to the drug addition (time 0). Bold lines represent the normalized average nuclear fluorescence. **B**) Increase in nuclear fluorescence of Trx-CFP and TrxR1-YFP is caused by CPT. Yellow and cyan lines correspond to the normalized average nuclear fluorescence of TrxR1-YFP and Trx-CFP, respectively. Full and open circles denote nuclear fluorescence in CPT- and mock-treated cells, respectively. Error bars represent standard error of at least three independent experiments. **C**) Cell-to-cell variability in nuclear fluorescence of both Trx-CFP and TrxR1-YFP decreases upon CPT addition. CV of nuclear fluorescence of TrxR1-YFP (yellow line), Trx-CFP (cyan line) and Cherry (red line) was calculated for 25h following CPT addition. Error bars denote standard error.

We found that upon CPT exposure both Trx-CFP and TrxR1-YFP gradually accumulated in the cell nuclei (see Movies S1 and S2, and Figure 4A). On average, CPT stimulation for 25h resulted in about 2-fold increase in nuclear levels of both proteins (1.78±0.1 for Trx-CFP and 1.95±0.12 for TrxR1-YFP). No significant CPT-induced nuclear accumulation was found for the Cherry-tagged proteins (See Movie S3 and Figure 4A). Likewise, in the control mock-treated cells no nuclear increase was observed for Trx-CFP and a small ∼1.2-fold increase was observed for TrxR1-YFP (Figure 4B).

No significant correlation (Spearman R<0.15) was found between the initial nuclear level (Fi) of either Trx-CFP or TrxR1-YFP and the amplitude of their nuclear accumulation following CPT addition (Fmax-Fi) (Figure S2A, B). Moderate anti-correlation was observed between the initial nuclear level (Fi) and the ratio of CPT-induced nuclear rise (Fmax/Fi) of either protein (R = −0.41, p<0.0001 for Trx and R = −0.3, p<0.0001 for TrxR1) (Figure S2C, D). The CV of nuclear levels for both proteins decreased upon CPT treatment by more than 30% (Figure 4C). Thus, CPT-induced nuclear translocation reduced the variability in nuclear levels of Trx/TrxR1 between cells.

In contrast to the nuclear accumulation observed following CPT addition, the total amounts of Trx-CFP and TrxR1-YFP did not vary significantly (Figure S3A), while the cytoplasmic levels of both proteins slightly decreased (Figure S3B). Also, no substantial changes in cell-to-cell variability in total amounts and cytoplasmic levels of the two proteins were observed (change in CV<20%) (Figure S3C, D).

We also measured nuclear enrichment defined as the ratio of nuclear fluorescence to total cell fluorescence. Similarly to the nuclear protein levels, nuclear enrichment increased in response to CPT by 2 fold for TrxR1-YFP and by 1.5 fold for Trx-CFP. The cell-to-cell variability in nuclear enrichment of both proteins decreased following CPT addition by more than 40% (Figure S4), again indicating that after CPT exposure the cells become more similar in their protein distribution profile.

### Trx-CFP and TrxR1-YFP are reliable reporters for nuclear accumulation of the wild-type endogenous Trx and TrxR1

It should be noted that the present study does not require the tagged proteins to be functional, but merely to be reliable reporters for the dynamics and localization of the endogenous proteins. As reported previously, the majority of endogenous proteins labeled internally with a fluorescent tag preserve their wild-type localizations and functions [17], [18], [19]. In our recent work we found that ∼80% of the endogenously tagged proteins from the LARC library serve as faithful markers for the dynamics of endogenous proteins [10]. In Trx-CFP/TrxR1-YFP cells the CFP tag is inserted between aa 63 and 64 of the Trx protein, leaving its catalytic site -CGPC- (aa 32–35) intact. The YFP tag is integrated between aa 3 and 4 of TrxR1, preceding the sites required for the TrxR1 catalytic activity.

We compared the dynamics of Trx-CFP from the Trx-CFP/TrxR1-YFP cells and of Trx-YFP from the LARC clone 160507pl1F3 (the YFP tag is inserted between Trx aa 8 and 9), in which TrxR1 is not tagged. Dynamics of CPT-induced nuclear enrichment for both Trx-CFP and Trx-YFP were quite similar (Figure S5A). Likewise, dynamics of TrxR1-YFP from either Trx-CFP/TrxR1-YFP cells or the parental LARC clone 010506pl1A12 or of TrxR1-YFP from the LARC clone 130207pl1E3, generated by an independent round of CD-tagging, were nearly identical (Figure S5B).

**Figure 5:**
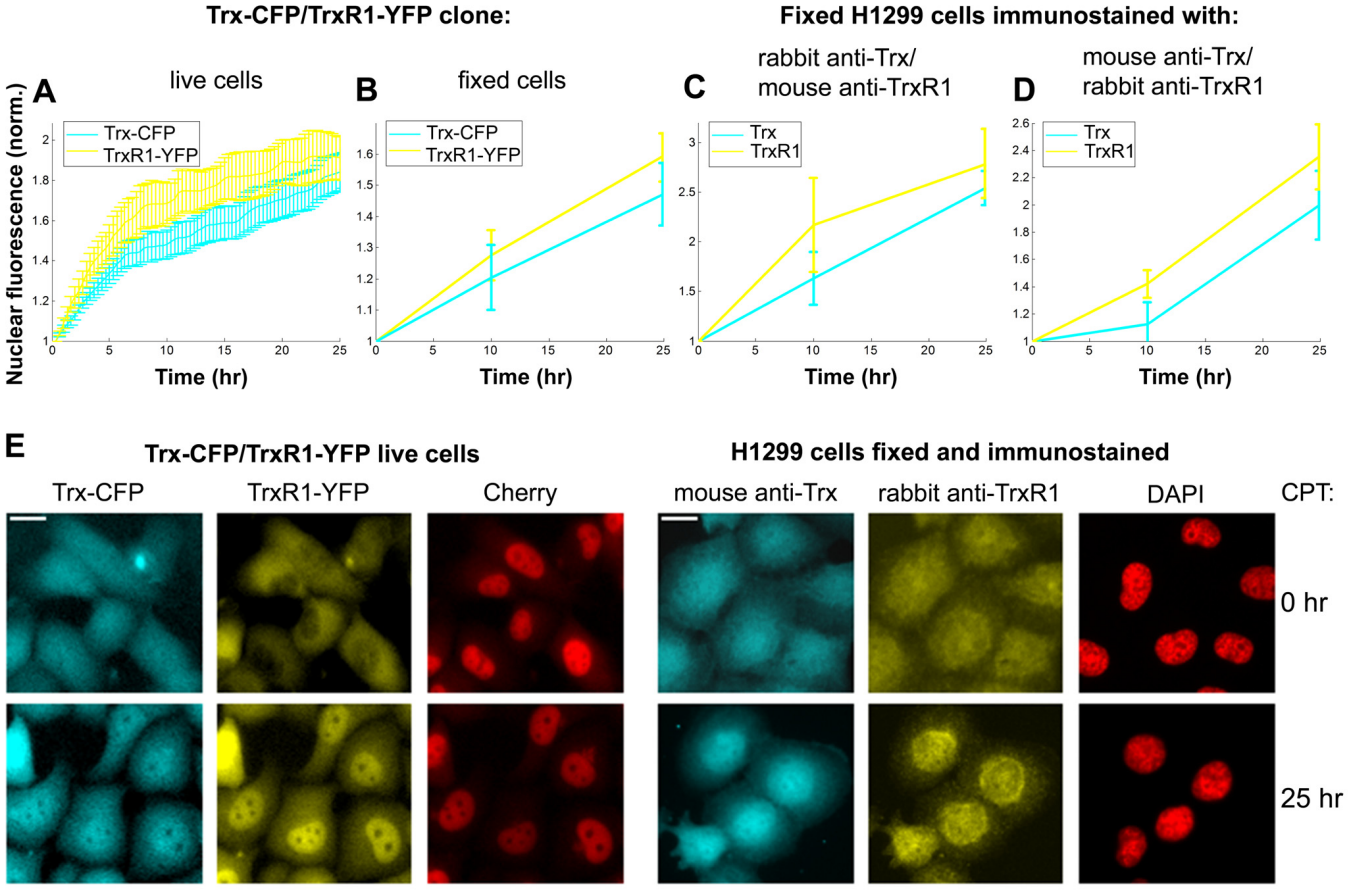
Both tagged and wild-type Trx and TrxR1 accumulate in the nucleus upon CPT treatment. **A**–**D**) Normalized average nuclear levels of both tagged and wild-type Trx (cyan lines) and TrxR1 (yellow lines) increase following CPT addition. Error bars are standard error of three independent experiments. **A**) Data from time-lapse movies of Trx-CFP/TrxR1-YFP cells. **B**) Data from images of Trx-CFP/TrxR1-YFP cells fixed at 0, 10 and 25 hours after CPT addition. **C**, **D**) Data from images of H1299 cells fixed at the same time points as above and immunostained with (**C**) rabbit anti-Trx and mouse anti-TrxR1 antibodies and (**D**) mouse anti-Trx and rabbit anti-TrxR1 antibodies. **E**) Fluorescent images of live Trx-CFP/TrxR1-YFP cells and of fixed and immunostained H1299 cells before (0h) and after (25h) CPT addition. Images of Trx-CFP/TrxR1-YFP cells represent snapshots from the time-lapse movie. DAPI, DAPI staining used to mark nuclear boundaries in the H1299 cells. Scale bars denote 20 microns.

We further tested whether the wild-type endogenous Trx and TrxR1 proteins localize to the nucleus upon CPT exposure. The H1299 cells expressing non-tagged Trx and TrxR1 were fixed at 0, 10 and 25 hours following CPT treatment and immunostained with two different pairs of anti-Trx and anti-TrxR1 antibodies. We found that following CPT addition both the wild-type Trx and TrxR1 proteins localized to the cell nuclei with dynamics similar to those observed for the fluorescently labeled Trx and TrxR1 (Figure 5).

**Figure 6:**
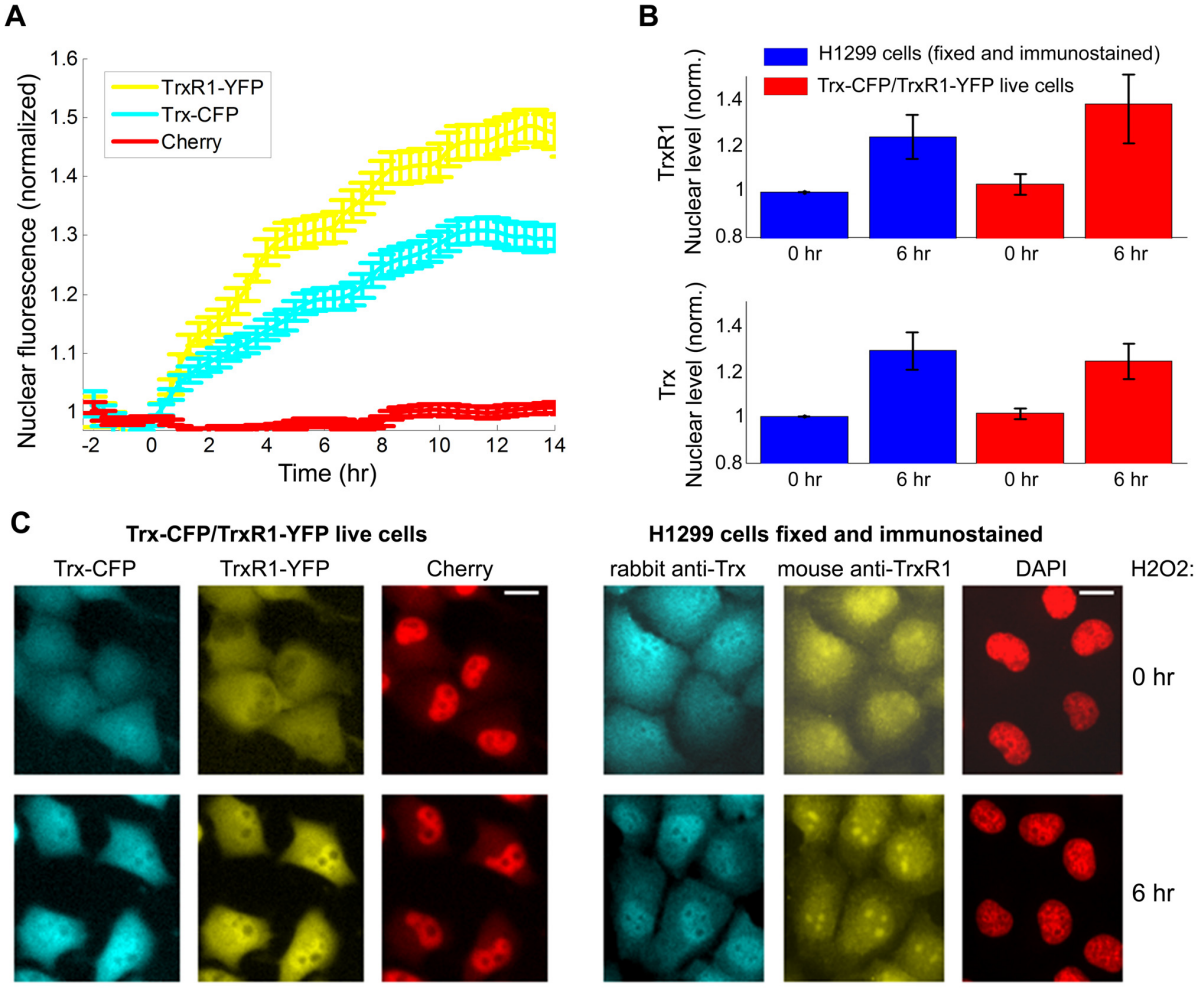
Nuclear levels of both tagged and wild-type Trx and TrxR1 increase after H_2_O_2_ exposure. **A**) Nuclear fluorescence profile of Trx-CFP, TrxR1-YFP and Cherry-tagged proteins following H_2_O_2_ addition. Data obtained from time-lapse movies of Trx-CFP/TrxR1-YFP cells. Error bars denote standard error. **B**) Nuclear accumulation of tagged and wild-type Trx and TrxR1 upon H_2_O_2_ treatment. Average nuclear protein levels measured before (0h) and after (6h) H_2_O_2_ addition. Blue bars, data from fixed H1299 cells immunostained for Trx and TrxR1. Red bars, data from time-lapse movies of live Trx-CFP/TrxR1-YFP cells. **C**) Fluorescent images of live Trx-CFP/TrxR1-YFP cells and of fixed H1299 cells immunostained for Trx and TrxR1 before (0h) and after (6h) H_2_O_2_ addition. Images of Trx-CFP/TrxR1-YFP cells represent snapshots from the time-lapse movie. DAPI staining is used to mark nuclear boundaries in the H1299 cells. Scale bars denote 20 microns.

We also treated the cells with H2O2, a classical inducer of oxidative stress. Using time-lapse microscopy we observed a considerable rise in nuclear levels of both Trx-CFP and TrxR1-YFP following H2O2 addition (Figure 6A). Nuclear accumulation was also observed in the H1299 cells fixed at 6 h after H2O2 exposure and immunostained for the endogenous Trx and TrxR1 proteins (Figure 6B, C).

Taken together, these results indicate that Trx-CFP and TrxR1-YFP are reliable reporters for dynamical localization changes of the wild-type Trx and TrxR1.

### Trx and TrxR1 show correlated dynamics

The basal levels of Trx and TrxR1 were substantially correlated (R = 0.64±0.05, p<0.0001 for total cell levels and R = 0.62±0.04, p<0.0001 for nuclear levels). Interestingly, nuclear enrichment showed the highest correlation (R = 0.87±0.02, p<0.0001). The correlation between Trx and TrxR1 was significantly higher than the correlation observed with the Cherry-tagged proteins (Figure S6).

**Figure 7:**
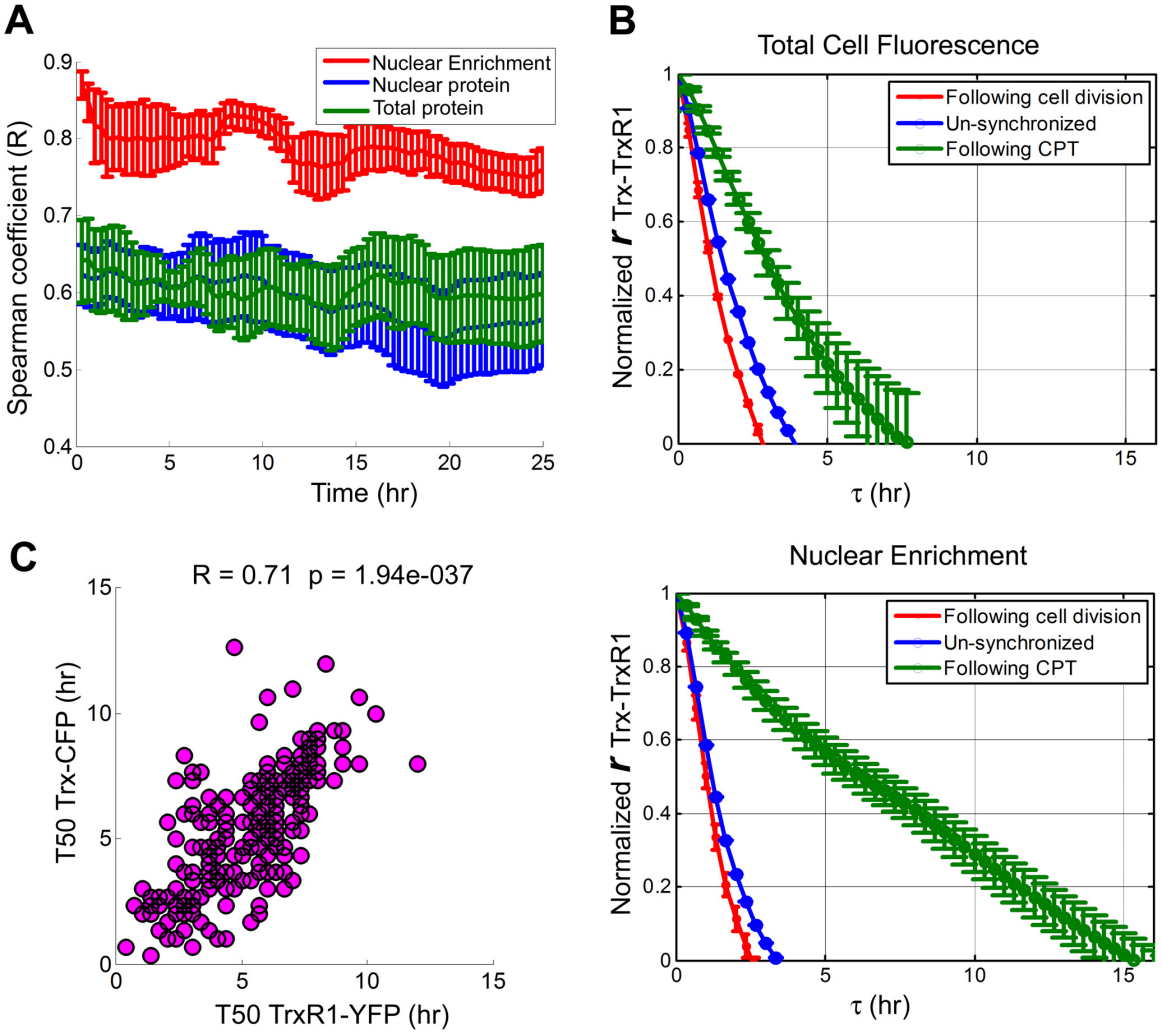
Trx-CFP and TrxR1-YFP are highly correlated in their response to CPT. **A**) Correlation of Trx-CFP and TrxR1-YFP following CPT addition: total cell fluorescence (green) and nuclear fluorescence (blue) of Trx-CFP and TrxR1-YFP show moderate correlation (minimal correlation values observed: R = 0.58, p<0.0001, for the total cell protein levels and R = 0.54, p<0.0001, for the nuclear protein levels), while nuclear enrichment (red) is highly correlated (minimal correlation value observed: R = 0.75, p<0.0001). Error bars denote standard error. **B**) Normalized cross correlation function of total cell protein levels (the upper panel) and of nuclear enrichment (the lower panel) of Trx-CFP and TrxR1-YFP in unsynchronized cells (blue lines), cell-cycle synchronized cells (red lines) and in cells exposed to CPT (green lines). Error bars denote standard error. **C**) T50 of Trx-CFP and TrxR1-YFP are highly correlated (R = 0.71 p<0.0001). T50 is defined as the time it takes for each protein to reach half of the total nuclear level observed 25 hours after CPT addition. Each circle in the scatter plot represents a measurement obtained from an individual cell. Altogether 233 cells were analyzed.

We next asked whether dynamics of Trx and TrxR1 in response to CPT are correlated as well. We found that during the 25 hours following CPT addition the correlation values observed for the basal protein levels slightly decreased (Figure 7A). Nuclear enrichment, which represents the dynamical changes in protein localization, remained the most correlative measure.

To further study the relationship of the Trx and TrxR1 dynamics, we calculated the cross correlation function 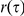 (see Methods) for both total cell fluorescence and nuclear enrichment under several conditions. The de-correlation time between the dynamics of the two proteins was defined as the time it takes for 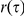 to decay by 50% (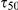). Longer de-correlation times represent stronger correlation between the two proteins. We found that under non-stimulated conditions (unsynchronized cells and cells synchronized to cell cycle *in-silico* (See Methods)) the decay time was relatively short: 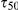 = 1.5–2 hours for both total cell protein fluorescence and nuclear enrichment measurements. However, upon CPT addition the decay time of the cross correlation increased to 3.5 hours for total cell fluorescence and to 6 hours for nuclear enrichment (Figure 7B). Thus, again, nuclear enrichment upon CPT treatment was the most correlative measure.

To further study the dynamical relationship of the two proteins we defined for each cell T_50_TrxR1-YFP and T_50_Trx-CFP as the time it takes for either protein to reach half of the nuclear level observed 25 hours following CPT addition. We found that T_50_TrxR1-YFP and T_50_Trx-CFP varied between cells in a way that was highly correlated (R = 0.71, p<0.0001; Figure 7C). Only mild correlation was found between either T_50_TrxR1-YFP or T_50_Trx-CFP and T_50_ of Cherry-tagged proteins (R≤0.23, p<0.001) (Figure S7). No dependence was found between the basal nuclear levels of each TrxR1-YFP and Trx-CFP and their T_50_ (Figure S8).

Taken together, these results imply that Trx and TrxR1 proteins respond in concert to CPT stimulation and that correlative aspects of their dynamics are even more prominent than those of their levels.

## Discussion

### A directed CD tagging method for fluorescent labeling of endogenous proteins

We presented a feasible approach for fluorescent tagging of endogenous proteins in human somatic cells. This is based on the rAAV-mediated directed targeting of an intronic region of the gene of interest with an artificial exon cassette containing a fluorescent protein-coding sequence. We designed a generic artificial exon CFP cassette, with one construct for each of three reading frames. To create a targeting construct, the CFP cassette in the desired reading frame is ligated between two regions of homology to the gene of interest, which are generated by PCR.

The successful knock-in of a fluorescent tag requires only one allele to be targeted, making it possible to tag endogenous proteins in both diploid and aneuploid cell lines. We applied directed CD tagging to label the thioredoxin 1 protein with CFP in the diploid HCT116 cell line (data not shown), and in the aneuploind H1299 cells.

A key feature of the present approach is that the fluorescent tag, introduced into the intronic sequence as an artificial exon, is expressed as a full-length fusion protein and serves as both a reporter tag and a promoterless selection marker. This feature offers several advantages. 1) It overcomes the need for a standard drug selection marker and its subsequent excision from the targeted gene locus by *cre* recombinase to restore the targeted allele to its natural configuration [36], [38], [63]. 2) The artificial exon fluorescent tag cassette can be introduced into almost any region of the intronic sequence of interest: the subsequent splicing into mRNA will result in correct in-frame fusion of the tag sequence to both flanking exons. The simplicity of this strategy contrasts with other knock-in tagging methods, where the tag sequence is directly fused to the endogenous exon, what requires customization of the junction point [37], [38], [39]. 3) The size of exogenous DNA that can be included into rAAV targeting vectors is limited by the packaging capacity of rAAV up to 4.8 kb [64]. To maximize targeting efficiency, homologous arms (HAs) must be as long as possible [65]. The standard drug selection cassettes are ∼2 kb in size. Inclusion of large tag sequences (such as fluorescent tag cassette, which is ∼0.8 kb in size) into rAAV vector, will leave a total space of only 2 kb for two HAs, translating to 1 kb per arm. Using fluorescent tag as a reporter and as a selection marker overcomes these size constrains and allows increasing the length of each HA up to 2 kb. We routinely use HAs that are 1.6–2 kb long. 4) Although the promoterless configuration of selection markers and utilization of rAAV as a DNA delivery tool significantly improved the efficiency of directed gene targeting, homologous recombination in human cells remains a very rare event. Therefore, the proportion of false-positive events introduced by either selection method is relatively high. We found that in our method only 3–10% of cells selected by FACS represent true CFP positive events, as detected by fluorescence microscopy. This is comparable to the percentages of recombinant (true-positive) clones detected among the drug-resistant colonies when a promoterless drug resistance marker was used for selection [34], [38]. Our method offers the advantage of rapid and easy visualization of recombinant clones by fluorescence microscopy, reducing the number of colonies utilized in the subsequent PCR-based screens to validate proper targeting, down to several clones (compared to several tens or even hundreds of clones that must be screened in the case of a standard drug selection [34], [37]).

In summary, the present directed tagging method is facile and can yield productive cell lines from rare but precise recombination events.

### Directed CD tagging can be used to establish multicolor reporter cell lines

Our method allows generation of reporter cell lines which express simultaneously several proteins of interest, where each protein is labeled endogenously with a different fluorescent tag. Creation of such reporter lines requires iterative rounds of directed CD tagging. At least three different fluorescent tags (e.g. red, yellow and cyan fluorophores) could be used simultaneously.

To generate double-labeled reporter cells we employed the H1299 LARC collection, which already contains over a thousand clones expressing different endogenous proteins labeled with YFP by non-directed CD tagging. Using cells from the LARC collection allows considerable shortening of the time required to generate double-labeled reporter lines, since only one round of directed tagging is required. We successfully applied this strategy to tag the Trx protein with CFP in the LARC clone expressing the TrxR1-YFP fusion protein. The resulting Trx-CFP/TrxR1-YFP reporter cells were utilized to study the dynamical relationship between Trx and TrxR1in individual living cells.

### Correlated dynamics and variability in the thioredoxin system

Here we provide a view of the thioredoxin system components, Trx and TrxR1, in individual living cells under basal conditions and following CPT treatment. Dynamics of Trx and TrxR1 proteins were followed at high temporal resolution and accuracy using Trx-CFP/TrxR1-YFP reporter cells. Endogenous tagging of both proteins preserved their native regulation, avoided overexpression concerns, and faithfully reported their nuclear entry and dynamical profiles.

We found substantial correlation between the levels of Trx-CFP and TrxR1-YFP in both the nucleus and the whole cell. These results are in line with previous observations demonstrating that proteins from the same biological systems tend to be highly correlated, whereas proteins from different systems are more weakly correlated [1], [66]. Interestingly, immunohistochemical studies of a large set of non-small lung carcinomas also revealed strong association between the expression levels of Trx and TrxR1 in both the nuclear and cytoplasmic compartments [59].

We further found that basal nuclear levels of both Trx-CFP and TrxR1-YFP showed high cell-to-cell variability. This variability is considerably higher than the variability observed for the total levels of either of the proteins and mainly stems from cells that are highly enriched in nuclear Trx-CFP and TrxR1-YFP even in the absence of stress. Such variability in subcellular distribution of both proteins within clonal cell population may be attributed, at least in part, to the differences in micro-environments inhabited by individual cells and/or variations in ROS levels within cells due to variations in internal states.

There are numerous reports demonstrating nuclear translocation of Trx in response to a variety of agents causing elevation of intracellular ROS [47], [48], [49], [50], [51], [53]. However, to the best of our knowledge, the changes in subcellular distribution of TrxR1 has not previously been reported. Here we find that following CPT treatment both Trx and TrxR1 accumulate in the cell nuclei. ROS-induced nuclear accumulation of Trx was previously shown to be dependent on karyopherin alpha [60]. Trx, which lacks nuclear localization signal (NLS), appears to interact with karyopherin alpha through lysines 81 and 82. Nuclear import of Trx is also suggested to be regulated by post-translation modification of Trx on cysteine 69 [60]. Thus, despite its small size (about 13 kDa), Trx does not seem to freely diffuse through the nuclear pores, but is rather imported into the nucleus in a regulated manner. Similarly to Trx, TrxR1 does not have NLS or NES motifs. The mechanism of nuclear accumulation of TrxR1 still remains to be elucidated.

Analysis of individual cells revealed no dependence between the basal nuclear levels of the two proteins and the rate of their nuclear accumulation upon CPT addition. Similarly, no sizable correlation was found between the basal nuclear levels and absolute amounts of Trx/TrxR1 entering the nucleus following CPT. Interestingly, a moderate anti-correlation between initial nuclear levels and the relative amount of Trx/TrxR1 protein entering the nucleus in response to CPT was observed. Put together with considerably reduced cell-to-cell variability in nuclear levels of both proteins after drug addition, these results might suggest a limit to the dynamic range of nuclear accumulation in CPT-treated cells for both Trx-CFP and TrxR1-YFP. The present data suggest that mechanism for Trx/TrxR1 nuclear accumulation differs from the “fold-change” response (where the intensity of response is proportional to the initial protein levels), which was observed in signaling proteins such as ERK2 [11] and beta-catenin [67]. The observed response of the thioredoxin system more resembles an “absolute” response mechanism.

The relative amount of protein located to the nucleus (nuclear enrichment) and the rate of nuclear accumulation following CPT addition are highly correlated between Trx and TrxR1 in individual cells. This correlation is more pronounced than the correlation observed for the absolute protein levels, indicating that dynamic behavior of both proteins in response to CPT is even more similar than their expression levels, and suggests a functional interdependence. This functional interdependence is also reflected by the slow decay rate of the cross correlation function calculated for nuclear enrichment of both proteins following CPT stimulation (in contrast to the fast decay rate observed in the absence of CPT).

The data above suggest that both proteins act in concert in response to stress, consistent with the fact that Trx and TrxR1 function together. Nuclear translocation of Trx is an important defense response when the cells are under unfavorable circumstances. It provides the necessary reducing equivalent to redox-sensitive transcription factors like NF-kappaB [52], [53], Ref-1/AP1 [54], Ref-1/p53 [55], Nrf2 [56] and HIF [57], which regulate the expression of stress-responsive genes. In this context, the concurrent nuclear accumulation of TrxR1 could promote regulation of these transcription factors by maintaining reduction of Trx *in situ*. This is supported by the notion that during oxidative stress caused by glucose and glutamine deprivation, Trx in the nuclear compartment is more protected against oxidation than in the cytoplasm [68].

The observed concurrent nuclear accumulation of both proteins suggests that nuclear translocation of Trx could be dependent on TrxR1 or vice versa. To test this hypothesis we attempted to knock down TrxR1 or Trx by means of siRNA. However, depletion of any one of the two proteins (Trx to ∼20% and TrxR1 to ∼35% of original protein level) did not alter the dynamics of nuclear translocation of either protein. Both proteins showed dynamics similar to those observed without the knockdown (data not shown). One possible explanation for this observation might be that the extent of knockdown was insufficient to alter protein behavior due to tremendous reserve capacity in vital redox systems. This possibility is supported by recent studies demonstrating that reduction of TrxR1 activity by more than 90% using either siRNA or inhibition with a gold compound aurothioglucose did not affect the redox state of Trx and its activity, suggesting that the remaining 10% of TrxR1 activity was sufficient to support reduction of Trx [69], [70]. Another possibility might be that nuclear translocation of both Trx and TrxR1 is dependent on an upstream regulator.

In summary, we presented an approach for targeted fluorescent tagging of endogenous proteins to generate multicolor reporter cell lines. This approach enables studies of dynamic behaviors of two proteins within individual living cells. We used this to study two proteins, Trx and TrxR1, constituting the thioredoxin system. We find that Trx and TrxR1 show large variability between cells in timing, localization and levels, but the two proteins vary between cells in a highly correlated manner. Further implementation of this approach would make it possible to study other systems and pathways in individual cells and eventually lead to construction of pathway reporter libraries to study different biological systems at the proteomics level.

## Materials and Methods

### Cells and cell culture reagents

The LARC library of YFP-tagged proteins in the H1299 non-small lung carcinoma cell line was previously described [10], [25]. In the present study we used the LARC clone 010506pl1A12 expressing the TrxR1-YFP fusion protein (www.dynamicproteomics.net). Cells were grown in RPMI1640 medium containing L-glutamine (21875, GIBCO) supplemented with 10% Fetal Calf Serum and 1% Penicillin-Streptomycin (Biological Industries, Israel).

The AAV-293 cells (Stratagene) were grown in high glucose DMEM medium (D5796, Sigma) supplemented as above.

### Construction of Trx-CFP rAAV targeting vector

#### 1. Construction of artificial exon CFP cassette

mCerulean coding sequence (with no start and stop codons) was PCR-amplified from the Addgene plasmid 15214 using forward and reverse primers that contained splice acceptor and donor sequences, respectively [71]. The primers also included EcoRV site at their 5′ termini. Primers' sequences are available upon request. Three separate PCR reactions, one for each reading frame, were performed. Each reading frame insert was then cloned into EcoRV site of the pBluescript II SK(+) vector (Stratagene).

#### 2. Generation of Trx Homologous Arms (HAs)

Two adjacent regions of homology to intron 3 of the thioredoxin 1 gene were PCR-amplified from genomic DNA obtained from H1299 cells, using Expand High Fidelity^Plus^ PCR System (Roche), as per manufacturer's instructions. PCR primers included restriction enzyme sites (underlined) compatible with the targeting vector assembly. The 1476 bp left HA was amplified using forward primer containing NotI site (ATAAGAATGCGGCCGCAAGTGCTGGGATTATCCACCACG) and reverse primer containing SalI site (ACCTGTCGACTCATACGGAAGTCCTCGAGTGC). The 1525 bp right HA was amplified using forward primer containing BamHI site (ACGCGGATCCAGAGCTCCTCCTTCATGTCTGC) and reverse primer containing NotI site (ATAAGAATGCGGCCGCTAGCCTGGCTAACACGGAGAAAC). HAs were then treated with appropriate pairs of restriction enzymes and subcloned into pBluescript II SK(+) vector for sequencing purposes.

#### 3. Assembling of targeting vector

The ITR-containing pAAV vector backbone was excised from the pAAV-MCS plasmid (Stratagene) by cleavage with NotI and subsequent dephosphorylation by calf intestinal alkaline phosphatase (NEB). The CFP cassette in reading frame 0 was excised from the pBluescript vector by treatment with SalI/BamHI. Left and right HAs were excised from the pBluescript vector by digestion with NotI/SalI and BamHI/NotI, respectively. The desired restriction fragments were gel-purified using 0.8% agarose gel electrophoresis. The DNA was recovered from the gel using the Qiagen Gel Extraction kit. Assembling of targeting construct composed of two Trx HAs, CFP cassette and pAAV vector backbone was accomplished by four-part ligation using the high-concentration T4 DNA Ligase (USB). Ligation product was purified by ethanol precipitation, dissolved in distilled water and electroporated into DH10B electrocompetent *E.coli* cells (Invitrogen). Recombinant clones were identified by colony-PCR using CFP-specific primers. The targeting vector DNA was prepared using the Qiagen Plasmid Maxiprep kit. Proper assembly of Trx-CFP rAAV targeting vector was further confirmed by restriction digestion analysis.

### Production of infectious rAAV stocks

Trx-CFP rAAV stocks were produced by co-transfection of the targeting vector described above (5.5 µg) and 5.5 µg of each pAAV-RC and pHelper plasmids (AAV Helper-Free System, Stratagene) into 70% confluent AAV-293 cells grown in 15 cm plates, using the JetPei™ transfection reagent (Polyplus transfection) as per manufacturer's instructions. rAAV particles were collected 3 days post-transfection by scraping cells from one 15 cm plate into 1 mL sterile PBS followed by 4 freeze/thaw cycles. Each cycle consisted of 10 min freeze in a dry ice/ethanol bath, and 10 min thaw in a 37°C water bath, vortexing after each thaw. The cell lysates were then clarified by centrifugation at 12K rpm in a benchtop microfuge to remove cell debris, and supernatant containing rAAV was used fresh or stored at −80°C.

In parallel with Trx-CFP rAAV stock preparation, control CFP rAAV stock was produced by co-transfection of pAAV-CFP reporter vector, pAAV-RC and pHelper into AAV-293 cells, as described above. pAAV-CFP construct directing expression of CFP from the CMV promoter was generated by cloning mCerulean coding sequence into multiple cloning site of pAAV-MCS plasmid. CFP fluorescence of transfected AAV-293 cells was monitored by fluorescence microscopy to estimate transfection efficiency.

### Infection of target cells

About 10^6^ cells from the TrxR1-YFP expressing LARC clone 010506pl1A12 were plated onto 10 cm dishes 24h before infection. At the time of infection, cell medium was aspirated and 6 ml of medium containing 1 mL of Trx-CFP rAAV stock was added to the cells. Twenty four hours later, infected cells were given fresh medium and grown for additional 2 days to increase probability of homologous recombination events. During this period, the cells were split into 15 cm plates to maintain the cell culture under logarithmic growth.

In parallel, TrxR1-YFP cells were infected with the control CFP rAAV stock to estimate infection efficiency. More than 75% of cells from the control infection were CFP-positive, as determined by FACS analysis (LSRII cell analyzer, Becton Dickinson).

Note that use of crude (low-titer) rAAV lysates is thought to lead to a very low probability of multiple genomic integration events in the same cell [36].

### Selection of recombinant cells by FACS and clone expansion

Following infection with Trx-CFP rAAV, the cells were harvested by trypsinization, washed twice with sterile calcium/magnesium-free PBS, re-suspended in cold PBS at ∼2×10^6^ cells/ml and filtered through a 50 µM mesh to generate a single-cell suspension. Afterwards, the cells were sorted on a FACSAriaII cell sorter (Becton Dickinson). Uninfected parental TrxR1-YFP cells were used as a negative control to determine the gating region to be used for selection of CFP-positive cells. Single CFP-positive recombinant cells were collected into individual wells of 384-well plastic optical plates (781091, Greiner Bio-One). The clones were expanded from single cells by incubating the plates for 10–14 days until the cells are confluent (the medium was changed every 3–4 days). During this period, the clones were further screened for CFP expression by fluorescence microscopy to detect true-positive events. Note that microscopic screen can also be performed following FACS sorting, immediately after the cells are spread out. However for the reasons of convenience and feasibility of the screen we recommend performing it after the cells form visible colonies. Confluent CFP-positive clones were then transferred from a 384-well plate to three 96 well plates and grown to confluence. Cells from two plates were frozen as previously described [25], cells from the third plate were used for 3′RACE and RT-PCR analyses to confirm proper targeting.

### 3′RACE

3′RACE analysis was performed essentially as described in Sigal *et al.* [25]. Total RNA was isolated from recombinant clones grown to confluence in a 96-well plate using ZR-96 mini RNA isolation I kit (Zymo Research). First-strand cDNA was synthesized from 1 µg total RNA using Omniscript RT Kit (Qiagen) and (dT)_17_-AP primer (GGCCACGCGTCGACTAGTAC(T)_17_). cDNA containing the CFP tag sequence was amplified by nested PCR using Ready-Mix PCR mix (Bio-Lab). The first PCR reaction was performed with AP primer lacking (dT)_17_ and mCerulean-specific primer f432 spanning nucleotides 432–451 (CAACGCCATCAGCGACAACG). The second (nested) PCR reaction was performed using the AP primer above and mCerulean-specific primer f606 spanning nucleotides 606–626 (GAGCACCCAGTCCAAGCTGAG). PCR products were purified by QIAquick PCR purification kit (Qiagen) and sequenced using the f606 primer.

### RT-PCR

Total RNA was isolated as described above. First-strand cDNA template was generated from 1 µg total RNA in a 20 µL reaction using oligo(dT)15 primer (Promega) and Omniscript RT Kit (Qiagen), as per manufacturer's instructions. Primer-specific cDNA was PCR-amplified using Ready-Mix PCR mix (Bio-Lab). mRNA expressed from both the CFP tagged and wild-type Trx alleles was detected using the forward primer f1Trx (AGCAGATCGAGAGCAAGACTG), which anneals in exon1 of Trx cDNA, and the reverse primer bTrx (CAGATGGCAACTGGGTTTATG), which anneals in exon 5. CFP-tagged Trx mRNA was detected using the forward primer f2Trx (AGTTGACTTCTCAGCCACGTG), which anneals in exon 2 of Trx cDNA, and the reverse primer bCFP (CTTGTACAGCTCGTCCATGC) annealing in the 3′ end of CFP sequence. Another primer pair used was the forward primer fCFP (GTGAGCAAGGGCGAGGAGCTG), which anneals in the 5′ end of CFP sequence and the reverse primer bTrx (see above).

### Immunoblot analysis

Total cell lysates from the control H1299 cell line, the parental TrxR1-YFP clone, and double-labeled Trx-CFP/TrxR1-YFP cells were prepared using RIPA buffer (Pierce) according to manufacturer's instructions. Protein concentrations were determined by BCA protein assay kit (Pierce). Total protein (15µg) was resolved on SDS-PAGE and transferred to Hybond-ECL nitrocellulose membrane (Amersham Biosciences). The membranes were then immunoblotted with anti-GFP, -Trx or -TrxR1 primary antibodies followed by HRP-conjugated secondary antibodies (Amersham Biosciences). Protein bands were visualized by ECL Western Blotting Detection Reagent (Amersham Biosciences). Intensity of protein bands was quantified using ImageJ software.

Mouse anti-GFP antibody (11814460001) was obtained from Roche and used at dilution 1∶600. The following antibodies were purchased from Santa Cruz: mouse anti-TrxR1 (sc-28321), rabbit anti-TrxR1 (sc-20147), mouse anti-Trx (sc-58441) and rabbit anti-Trx (sc-20146). Mouse antibodies were used at dilution 1∶150, rabbit antibodies were diluted 1∶250.

### Cell treatment with CPT and H2O2

Camptothecin (CPT, C9911, Sigma) was dissolved in DMSO (D2650, Sigma) to obtain a stock solution of 10mM. In each experiment the drug was diluted to 10 µM in complete cell medium, and cell growth medium was replaced by the diluted drug. In time-lapse movies, the medium (2mL) was replaced under the microscope after at least 5 rounds of images taken before drug addition.

Hydrogen Peroxide solution (H2O2, 30%) was obtained from J.T.Baker. To prepare stock solution, H2O2 was diluted 1∶500 in sterile PBS. Stock concentration was determined by a spectrophotometric method as described previously [72]. H2O2 concentration in the stock solution was about 20 mM. Immediately before addition to the cell culture, H2O2 stock was diluted in complete cell medium to a final concentration of 80 µM. Cell treatment with H2O2 was carried out as described above.

### Immunofluorescence

H1299 cells grown on fibronectin-coated coverslips were fixed with 3% paraformaldehyde in PBS for 20 min at room temperature and permeabilized with 0.5% Triton X-100 in PBS for 5 min. The cells were then blocked for 1h with 2% BSA in PBS and incubated for 1h with primary antibodies diluted 1∶100 in 2% BSA/PBS. Two pairs of primary antibodies (Santa Cruz) were used: mouse anti-Trx (sc-58441)/rabbit anti-TrxR1 (sc-20147) and rabbit anti-Trx (sc-20146)/mouse anti-TrxR1 (sc-28321). After washing in PBS the cells were incubated for 1h with fluorchrome-conjugated secondary antibodies: Cy3-conjugated goat anti-rabbit (1∶400, Jackson Immunoresearch) and Alexa Fluor 488-conjugated goat anti-mouse (1∶200, Molecular Probes). 4′-6-Diamidino-2-phenylindole-2HCl (DAPI, D9542, Sigma) was used at a concentration of 0.05µg/mL for 30 min as a nuclear counterstain. The cells were then washed with PBS and mounted in Elvanol (Mowiol 4–88, Hoechst, Germany). Images were acquired with Leica DMIRE2 inverted fluorescence microscope at 20× magnification using appropriate filter sets. Image analysis was performed with custom-written software described below. In each experiment, about 500 cells were analyzed per condition.

### Time-lapse microscopy

Time-lapse movies were obtained at 20× magnification as described in Sigal *et al.* [28] with an automated, incubated (including humidity and CO2 control) Leica DMIRE2 inverted fluorescence microscope and an ORCA ER cooled CCD camera (Hamamatsu Photonics). The system was controlled by ImagePro5 Plus (Media Cybernetics) software which integrated time-lapse acquisition, stage movement, and software based auto-focus. During the experiment, cells were grown and visualized in 12-well coverslip bottom plates (MatTek) coated with 10µM fibronectin (Sigma). The standard RPMI medium was replaced with RPMI without phenol red (Biological Industries, Israel) to decrease autofluorescence. For each well, time-lapse movies were obtained at four fields of view. Each movie was taken at a time resolution of 20 minutes and was filmed for at least 30 hours (over 90 time points). Each time point included four images: phase contrast; red, yellow and cyan fluorescence. For an example see Movies S1, S2, S3, S4.

### Image analysis of time-lapse movies

We used custom image analysis software described in Cohen *et al.* [10] with some modifications to allow measurements of 3 different fluorescent colors. The main steps in this software include: background correction (flat field correction and background subtraction), cellular and nuclear segmentation, cell tracking, and automated identification of cell division events. No significant bleaching was observed (on average less than 3% over the duration of the experiment). Cell and nuclei segmentation was based on the red fluorescent images of mCherry-tagged proteins common to all LARC clones, whose expression creates fluorescence pattern which is bright in the nucleus and dimmer in the cytosol and is relatively uniform across the cells. Global image threshold computed according to Otsu's method [73] was used to differentiate between cell boundaries and background. Segmentation of neighboring cells was performed by applying the seeded watershed segmentation algorithm. Seeds were obtained by smoothening of the red intensity image and usage of the nuclei as cell seeds. For nuclei segmentation red fluorescence intensity of each cell was stretched between zero and one and a fixed threshold was used to differentiate between the nucleus and the cytoplasm. Cell tracking was performed by analysis of the movie from end to start and linking each segmented cell to the cell in the previous image with the closest centroid. Cell division events were identified automatically using *in-silico* synchronization as described in Sigal *et al.* [28]. In short, cell division was detected by a sharp twofold drop in total fluorescence level between consecutive images.

### Comparison of tagged and parental clones

We compared the morphology, cell cycle duration and motility of the precursor H1299 cells tagged with Cherry only, the parental TrxR1-YFP cells and the Trx-CFP/TrxR1-YFP double tagged cells, using time-lapse microscopy. We find that the motility (mean velocity of center of mass) of H1299-Cherry, TrxR1-YFP and Trx-CFP/TrxR1-YFP cells was 8.6±0.5, 8.2±0.5 and 8.4±0.6 microns/hour, respectively (the data represent mean ± standard error); duration of cell-cycle was 27±4, 29±5 and 29±3 hours, respectively, and the clones had undistinguishable cell morphology.

### Cross correlation calculation

The cross correlation function 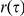 was calculated using the following equation:
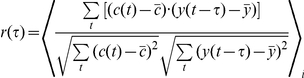

*c(t)* and *y(t)* denote measurements of CFP and YFP fluorescence, respectively, at time t in an individual cell; 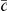 and 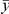 denote the average CFP and YFP fluorescence at time t, and 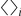 denote average over cells. 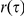 was calculated for total cell fluorescence and nuclear enrichment in unsynchronized cells, cells synchronized to the cell-cycle *in silico*, and CPT treated cells.

## Supporting Information

Figure S1 Normalized basal distributions of whole cell levels (A) and nuclear levels (B) of Trx-CFP (cyan line), TrxR1-YFP (yellow line) and Cherry (red line). Protein distribution profiles were normalized to 1 by dividing the protein level of each cell by the average protein level of all cells. A fraction of cells highly enriched in nuclear Trx-CFP/TrxR1-YFP is represented by a long right tail. Error bars denote standard error.
(0.36 MB TIF)

Figure S2 A–B) No correlation is observed between the initial nuclear level (Fi) of TrxR1-YFP (A) or Trx-CFP (B) and the absolute amount of protein entering the nucleus upon CPT addition. The absolute amount is defined as the difference between the maximal (within 25 hours following CPT addition) and initial nuclear levels (Fmax-Fi) of either protein. C–D) Moderate anti-correlation is observed between Fi and the relative amount of protein entering nucleus (Fmax/Fi). For TrxR1 R = −0.3 p<0.0001, Trx R = −0.41, p<0.0001. Robustness of correlation values was checked by bootstrapping. Each circle in the scatter plot represents a measurement obtained from an individual cell for TrxR1-YFP (yellow circles) or for Trx (cyan circles). Altogether 303 cells were analyzed.
(1.39 MB TIF)

Figure S3 Total cell levels of Trx-CFP and TrxR1-YFP do not change significantly upon CPT addition, while cytoplasmic levels slightly decrease. Normalized average total cell fluorescence (A) and cytoplasmic fluorescence (B) profiles of Trx-CFP (cyan line) and of TrxR1-YFP (yellow line) are shown. Cell-to-cell variability in total cell fluorescence (C) and cytoplasmic fluorescence (D) of both Trx-CFP and TrxR1-YFP shows no considerable changes following CPT addition. CV, coefficient of variance. Error bars denote standard error of three independent experiments.
(0.91 MB TIF)

Figure S4 A) Nuclear enrichment of both Trx-CFP (cyan line) and TrxR1-YFP (yellow line) increases after CPT addition. B) CV of nuclear enrichment for both Trx-CFP and TrxR1-YFP decreases following CPT addition. Error bars represent standard error.
(1.27 MB TIF)

Figure S5 Trx and TrxR1 proteins labeled with a fluorescent tag at different locations show similar dynamics of nuclear accumulation upon CPT treatment. A) Trx nuclear enrichment. Blue line, average nuclear enrichment of Trx-CFP from the Trx-CFP/TrxR1-YFP clone (CFP is inserted into intron 3 of the thioredoxin gene); red line, average nuclear enrichment of Trx-YFP from the LARC clone 160507pl1F3 (YFP is inserted into intron 1 of the thioredoxin gene). B) TrxR1 nuclear enrichment. Red line, average nuclear enrichment of TrxR1-YFP from the Trx-CFP/TrxR1-YFP clone; blue line, average nuclear enrichment of TrxR1-YFP from the parental LARC clone 010506pl1A12; black line, average nuclear enrichment of TrxR1-YFP from the LARC clone 130207pl1E3, generated by an independent round of CD-tagging. Error bars denote standard error.
(1.45 MB TIF)

Figure S6 Correlation between protein levels of Trx and TrxR1 is significantly higher than that observed with the Cherry-tagged (control) proteins. Correlations between total cell levels, nuclear levels and nuclear enrichment of Trx-CFP, TrxR1-YFP and Cherry are shown. Black bars represent correlation between Trx and TrxR1, grey bars- between Trx and Cherry, white bars- between TrxR1 and Cherry. Error bars denote standard error. P-values are less than 0.001.
(0.24 MB TIF)

Figure S7 Low correlation is found between (A) T50 of Trx-CFP and Cherry (R = 0.23 p<0.001) and (B) T50 of TrxR1-YFP and Cherry (R = 0.20 p<0.001). T50 is defined as the time it takes for each protein to reach half of the total nuclear level observed 25 hours after CPT addition. Each circle in the scatter plot represents a measurement obtained from an individual cell. Altogether 233 cells were analyzed.
(0.88 MB TIF)

Figure S8 No correlation is observed between basal nuclear levels of either protein (Trx-CFP or TrxR1-YFP) and rate of their nuclear accumulation following CPT. Rate of nuclear accumulation is denoted as T50.
(1.12 MB TIF)

Movie S1 Time-lapse movie of cyan fluorescence images of Trx-CFP/TrxR1-YFP reporter cells. Movie duration is 38 hours, 2 hours before drug addition followed by 36 hours after drug addition (time-lapse: 1 frame per 20 min).
(4.53 MB AVI)

Movie S2 Time-lapse movie of yellow fluorescence images of Trx-CFP/TrxR1-YFP reporter cells. Movie duration is 38 hours, 2 hours before drug addition followed by 36 hours after drug addition (time-lapse: 1 frame per 20 min).
(4.17 MB AVI)

Movie S3 Time-lapse movie of red fluorescence images of Trx-CFP/TrxR1-YFP reporter cells. Movie duration is 38 hours, 2 hours before drug addition followed by 36 hours after drug addition (time-lapse: 1 frame per 20 min).
(1.99 MB AVI)

Movie S4 Time-lapse movie of transmitted light images of Trx-CFP/TrxR1-YFP reporter cells. Movie duration is 38 hours, 2 hours before drug addition followed by 36 hours after drug addition (time-lapse: 1 frame per 20 min).
(5.17 MB AVI)
