# Supplementary figures and images for: Generation of Double-Labeled Reporter Cell Lines for Studying Co-Dynamics of Endogenous Proteins in Individual Human Cells
Source: PLoS One. 2010 Oct 21;5(10):e13524. doi: 10.1371/journal.pone.0013524 (PMC2958823; doi:10.1371/journal.pone.0013524)

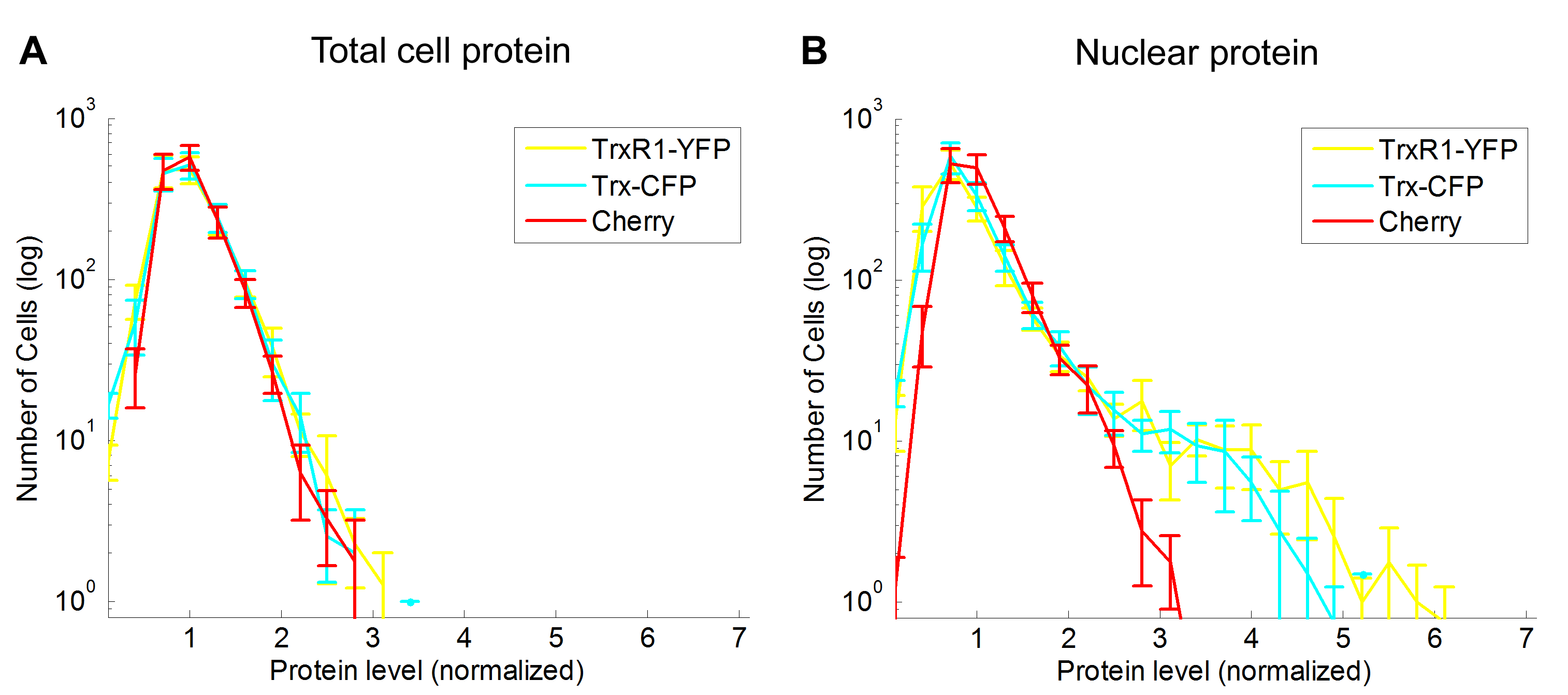

Supplement: Figure S1 — Normalized basal distributions of whole cell levels (A) and nuclear levels (B) of Trx-CFP (cyan line), TrxR1-YFP (yellow line) and Cherry (red line). Protein distribution profiles were normalized to 1 by dividing the protein level of each cell by the average protein level of all cells. A fraction of cells highly enriched in nuclear Trx-CFP/TrxR1-YFP is represented by a long right tail. Error bars denote standard error. (0.36 MB TIF) [file pone.0013524.s001.tif]

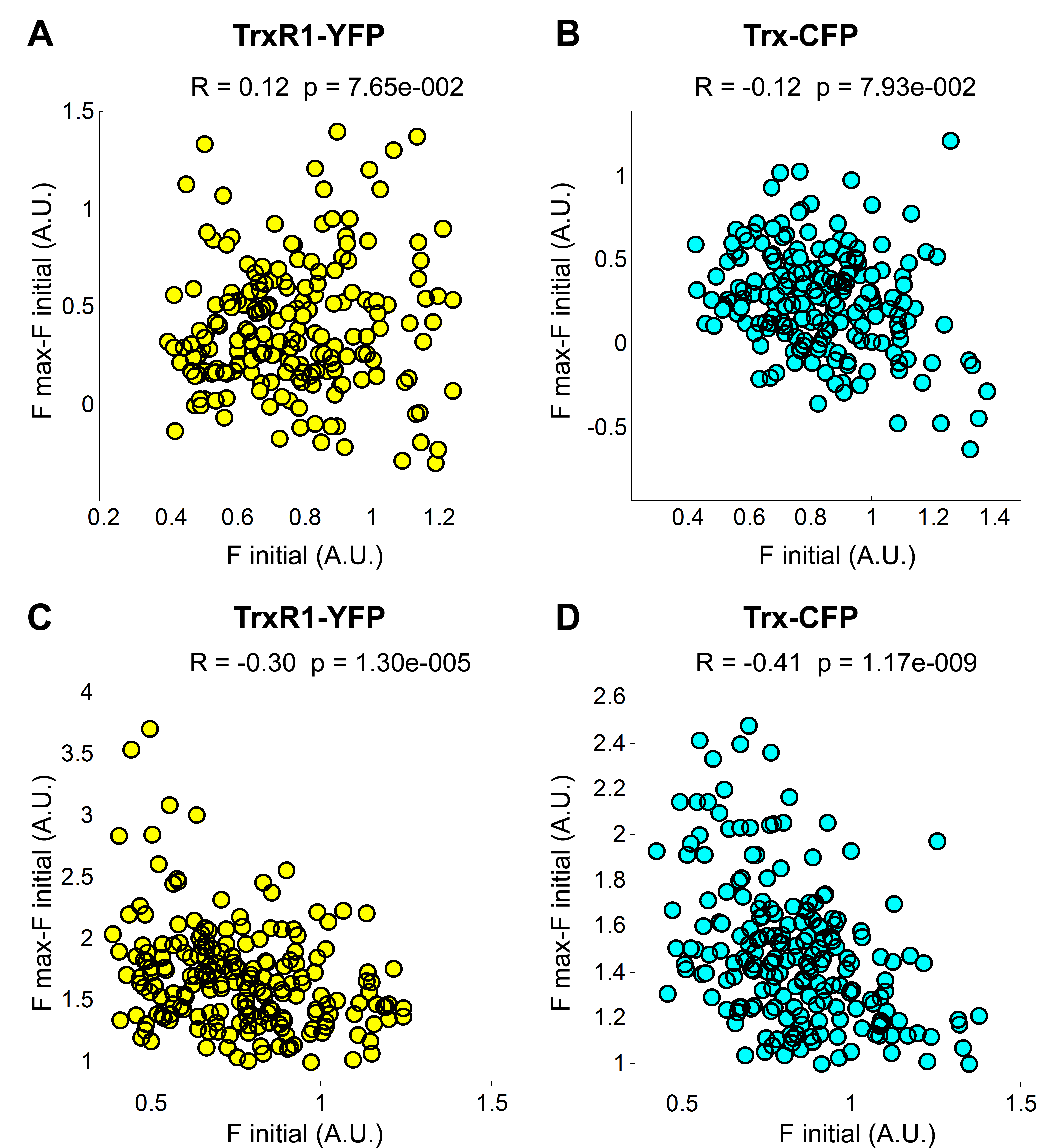

Supplement: Figure S2 — A–B) No correlation is observed between the initial nuclear level (Fi) of TrxR1-YFP (A) or Trx-CFP (B) and the absolute amount of protein entering the nucleus upon CPT addition. The absolute amount is defined as the difference between the maximal (within 25 hours following CPT addition) and initial nuclear levels (Fmax-Fi) of either protein. C–D) Moderate anti-correlation is observed between Fi and the relative amount of protein entering nucleus (Fmax/Fi). For TrxR1 R = −0.3 p<0.0001, Trx R = −0.41, p<0.0001. Robustness of correlation values was checked by bootstrapping. Each circle in the scatter plot represents a measurement obtained from an individual cell for TrxR1-YFP (yellow circles) or for Trx (cyan circles). Altogether 303 cells were analyzed. (1.39 MB TIF) [file pone.0013524.s002.tif]

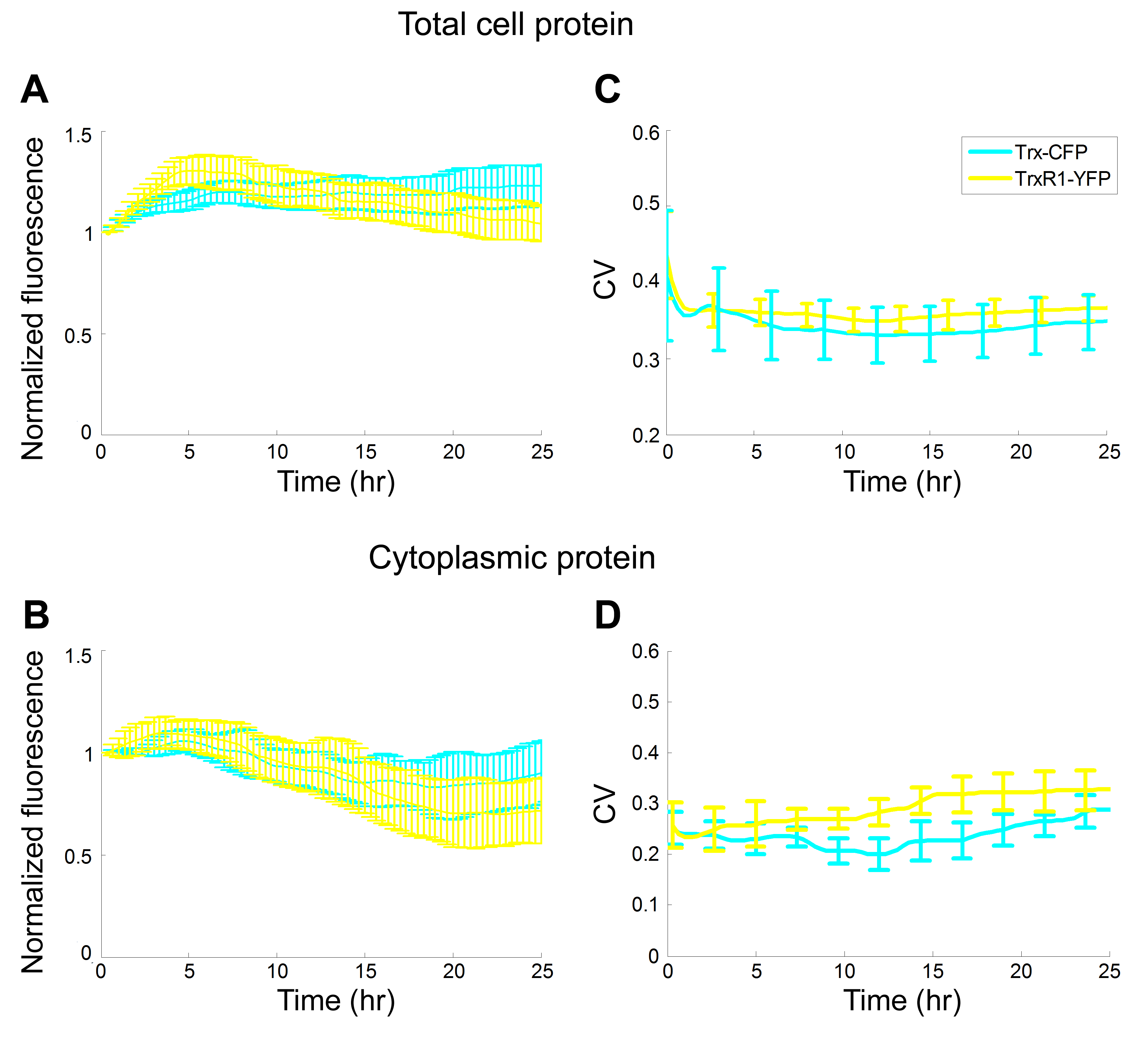

Supplement: Figure S3 — Total cell levels of Trx-CFP and TrxR1-YFP do not change significantly upon CPT addition, while cytoplasmic levels slightly decrease. Normalized average total cell fluorescence (A) and cytoplasmic fluorescence (B) profiles of Trx-CFP (cyan line) and of TrxR1-YFP (yellow line) are shown. Cell-to-cell variability in total cell fluorescence (C) and cytoplasmic fluorescence (D) of both Trx-CFP and TrxR1-YFP shows no considerable changes following CPT addition. CV, coefficient of variance. Error bars denote standard error of three independent experiments. (0.91 MB TIF) [file pone.0013524.s003.tif]

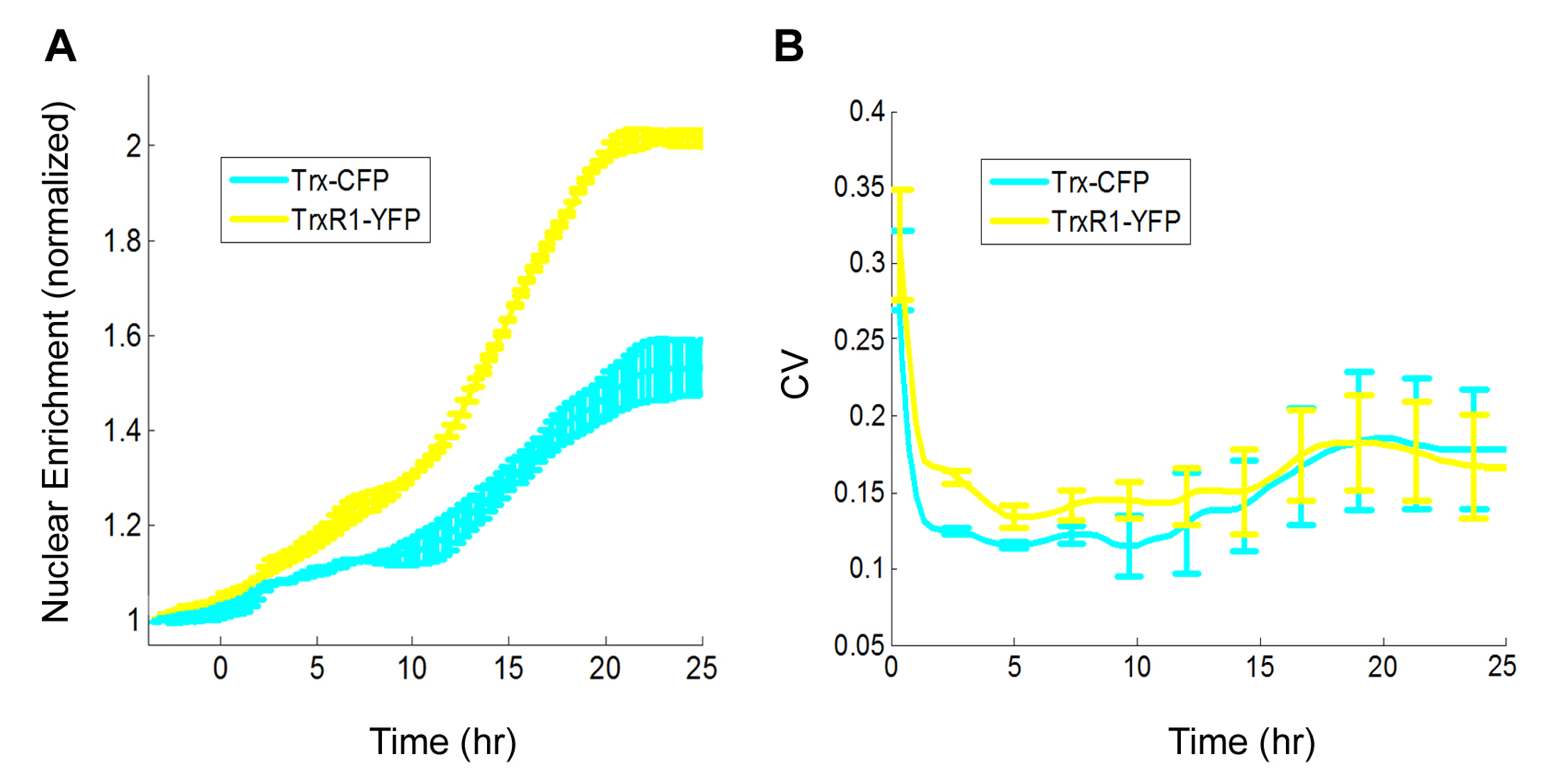

Supplement: Figure S4 — A) Nuclear enrichment of both Trx-CFP (cyan line) and TrxR1-YFP (yellow line) increases after CPT addition. B) CV of nuclear enrichment for both Trx-CFP and TrxR1-YFP decreases following CPT addition. Error bars represent standard error. (1.27 MB TIF) [file pone.0013524.s004.tif]

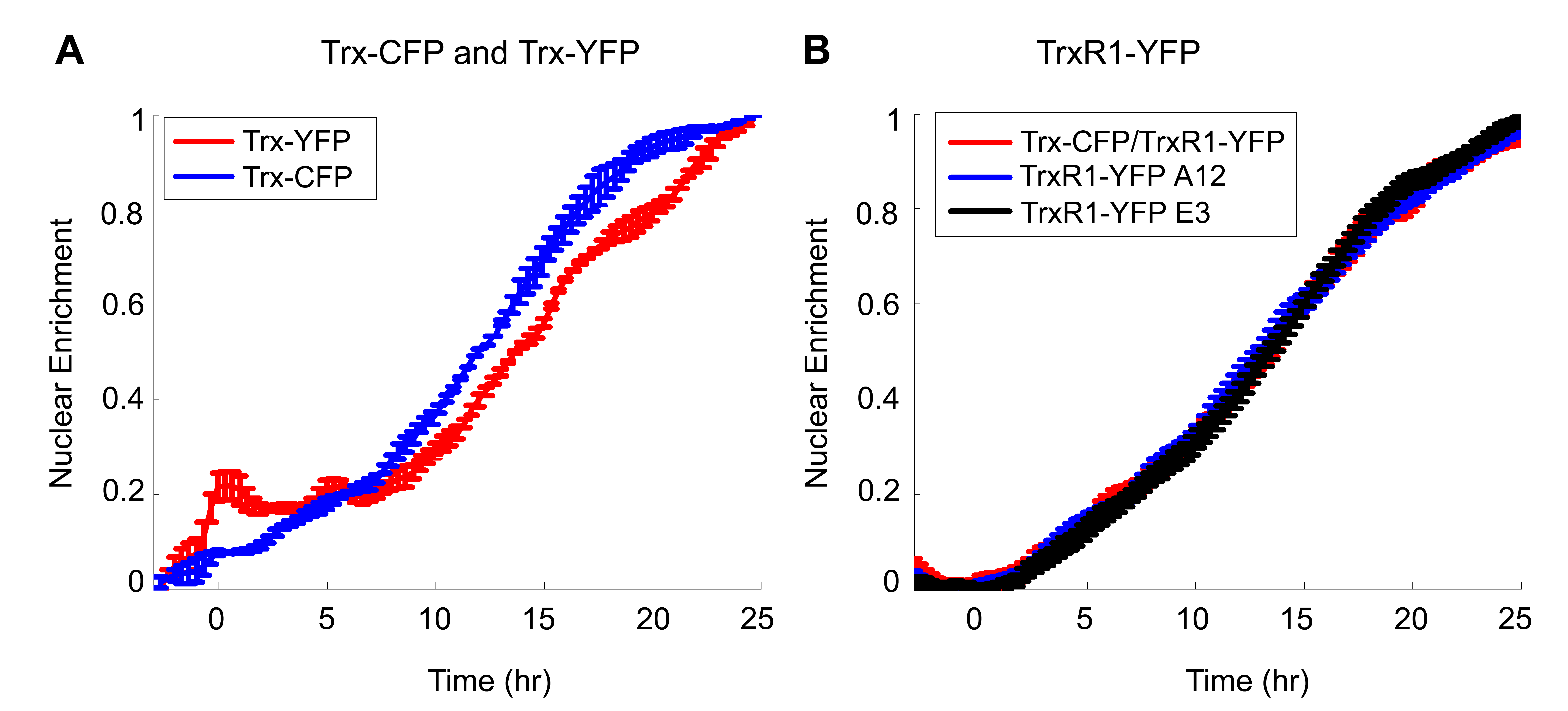

Supplement: Figure S5 — Trx and TrxR1 proteins labeled with a fluorescent tag at different locations show similar dynamics of nuclear accumulation upon CPT treatment. A) Trx nuclear enrichment. Blue line, average nuclear enrichment of Trx-CFP from the Trx-CFP/TrxR1-YFP clone (CFP is inserted into intron 3 of the thioredoxin gene); red line, average nuclear enrichment of Trx-YFP from the LARC clone 160507pl1F3 (YFP is inserted into intron 1 of the thioredoxin gene). B) TrxR1 nuclear enrichment. Red line, average nuclear enrichment of TrxR1-YFP from the Trx-CFP/TrxR1-YFP clone; blue line, average nuclear enrichment of TrxR1-YFP from the parental LARC clone 010506pl1A12; black line, average nuclear enrichment of TrxR1-YFP from the LARC clone 130207pl1E3, generated by an independent round of CD-tagging. Error bars denote standard error. (1.45 MB TIF) [file pone.0013524.s005.tif]

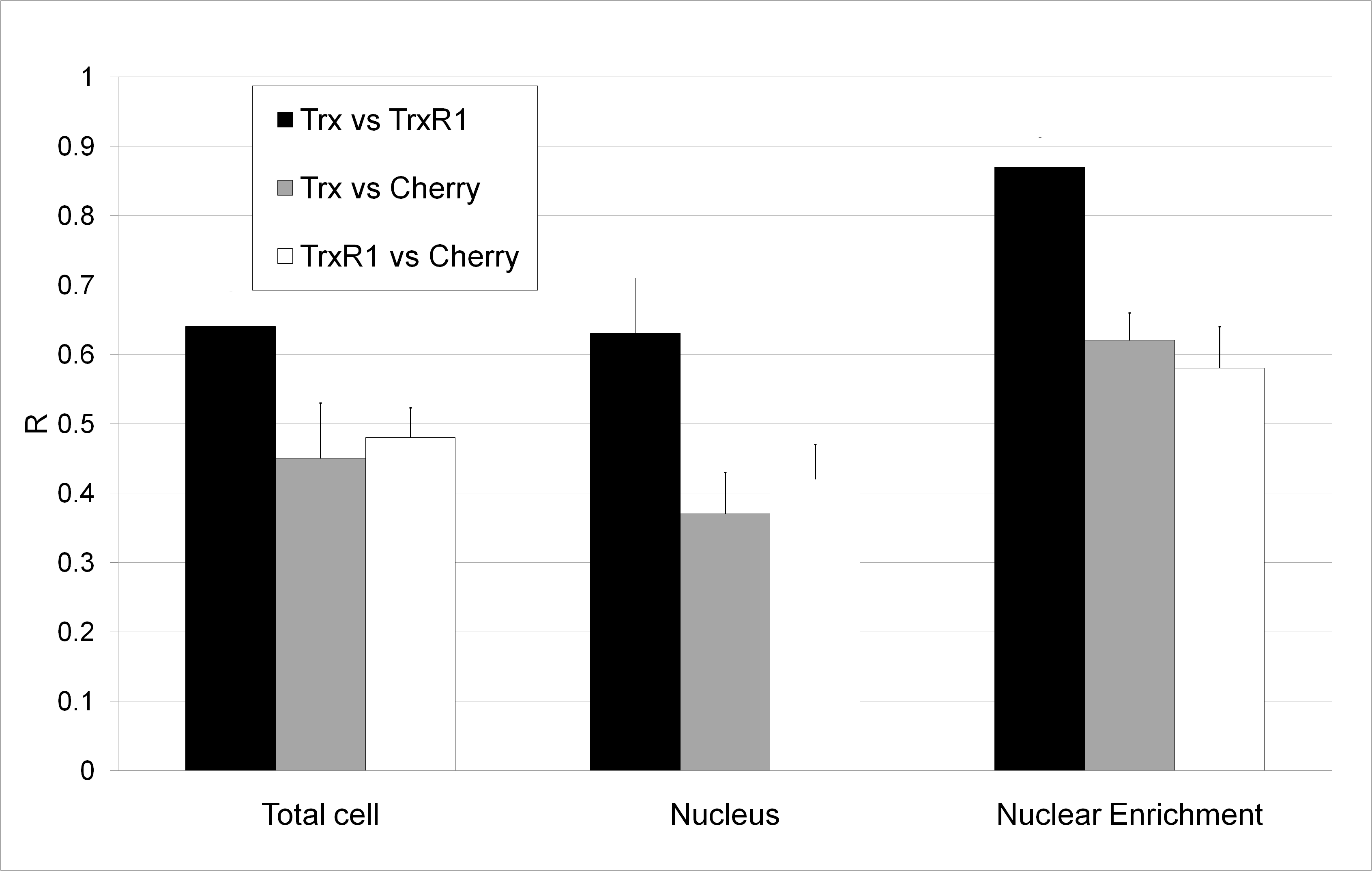

Supplement: Figure S6 — Correlation between protein levels of Trx and TrxR1 is significantly higher than that observed with the Cherry-tagged (control) proteins. Correlations between total cell levels, nuclear levels and nuclear enrichment of Trx-CFP, TrxR1-YFP and Cherry are shown. Black bars represent correlation between Trx and TrxR1, grey bars- between Trx and Cherry, white bars- between TrxR1 and Cherry. Error bars denote standard error. P-values are less than 0.001. (0.24 MB TIF) [file pone.0013524.s006.tif]

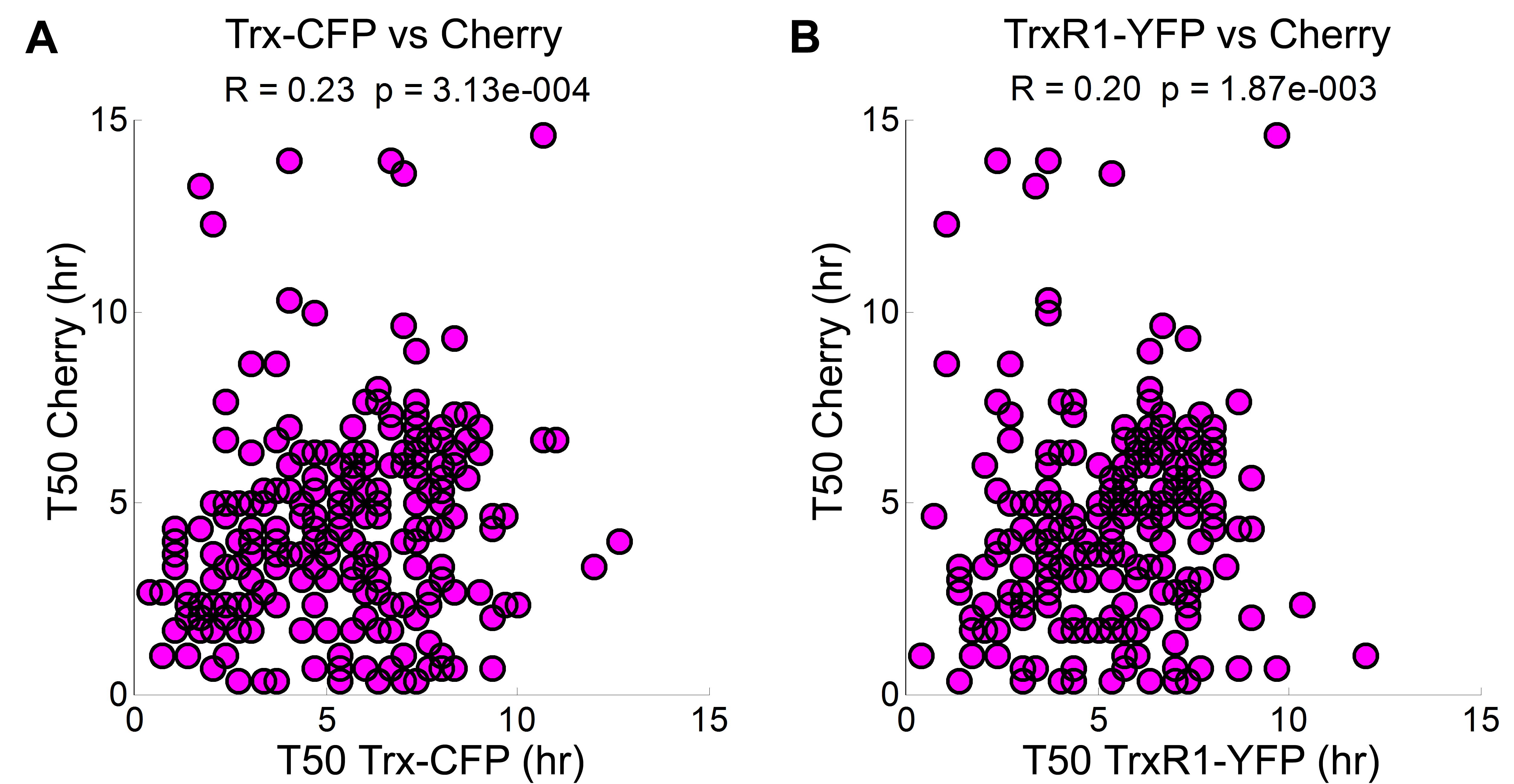

Supplement: Figure S7 — Low correlation is found between (A) T50 of Trx-CFP and Cherry (R = 0.23 p<0.001) and (B) T50 of TrxR1-YFP and Cherry (R = 0.20 p<0.001). T50 is defined as the time it takes for each protein to reach half of the total nuclear level observed 25 hours after CPT addition. Each circle in the scatter plot represents a measurement obtained from an individual cell. Altogether 233 cells were analyzed. (0.88 MB TIF) [file pone.0013524.s007.tif]

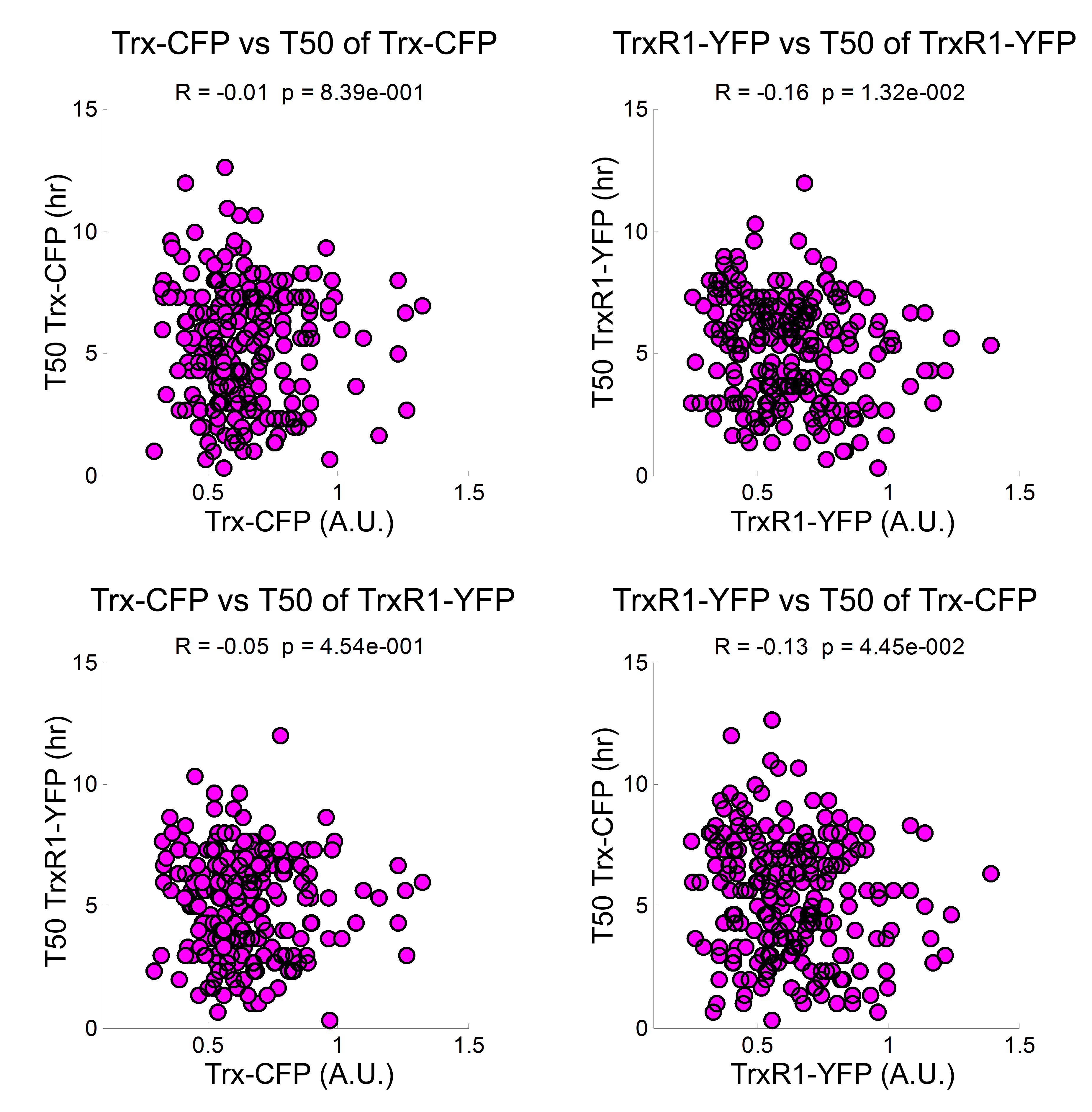

Supplement: Figure S8 — No correlation is observed between basal nuclear levels of either protein (Trx-CFP or TrxR1-YFP) and rate of their nuclear accumulation following CPT. Rate of nuclear accumulation is denoted as T50. (1.12 MB TIF) [file pone.0013524.s008.tif]
